# Insights into In Situ Benthic Caging Tests for Ecotoxicity Assessments Targeting Discharging Groundwater Contaminant Plumes

**DOI:** 10.1007/s00244-024-01075-9

**Published:** 2024-07-03

**Authors:** J. W. Roy, L. Grapentine

**Affiliations:** Water Science and Technology Directorate, Environment and Climate Change Canada, Burlington, ON Canada

## Abstract

While in situ toxicity testing with caged organisms has been used to assess surface water and sediment contamination, no successful application to benthic organisms exposed to highly contaminated groundwater plumes discharging to surface waters has been reported. The objective of this study was to demonstrate and evaluate this application using four sets of tests performed at three previously reported contaminated groundwater sites, which include one river site affected by volatile organic contaminant plumes, and two sites, one pond and one small urban stream, impacted by landfill plumes. The study examined multiple cage designs and orientations and two test organisms: an amphipod (*Hyalella azteca*) and midge larvae (*Chironomus riparius*; only one study). Cages were deployed for between 5 and 28 days and assessed for organism survival and growth. At all sites and for some deployment conditions, cages exposed to high contaminant concentrations in the plume footprint had greater mortality compared to those exposed to lower or background concentrations. Organism growth was less clear as a metric of toxicity. Vertically oriented cages typically showed high mortality to plume contaminants, but some were also affected by other non-target groundwater conditions (e.g., low dissolved oxygen, other contaminant sources), while horizontally oriented cages were rarely responsive to either groundwater influence. A hybrid cage design showed much promise in its single study. Useful observations on the test organisms and on potentially problematic site conditions were also made. The informed use of in situ toxicity cages could be an additional beneficial tool for groundwater contaminated site assessments.

**Supplementary Information:**

The online version contains supplementary material available at 10.1007/s00244-024-01075-9.

Human activities can cause groundwater contamination by a host of inorganic and organic chemicals, with subsequent groundwater transport of mobile and persistent contaminants to surface waters being a well-documented problem (Conant et al. 2019). Within the groundwater contaminant discharge zone, aquatic organisms living in or on the bottom sediments (benthic zone) can be exposed to high chemical concentrations (e.g., Hua et al. 2023). An assessment of the risk this poses to aquatic life of the benthic zone commonly involves comparing contaminant concentrations measured in a sample of discharging groundwater, or of the receiving surface water in some cases, to that of regulated water quality guidelines (WQG; objectives, standards, etc.). However, this approach has its drawbacks (see Roy et al. 2019). For instance, the WQG may not be highly applicable to the groundwater matrix or targeted to benthic organisms specifically. Also, WQG may not be appropriate for complex mixtures, which are common at contaminated groundwater sites, while some contaminants have no WQG currently. In addition, collecting water samples that properly capture the environmental conditions and actual exposure at and near the sediment interface in groundwater contaminant discharge areas can be difficult.

An alternate option for assessing the contaminant risk to the sediment interface zone is the use of in situ toxicity testing using caged organisms (US EPA 1994), a method that has been applied to aquatic environments for > 4 decades (e.g., 1984 study in Grapentine and Rosenberg 1992). These tests involve placing exposure cages (chambers with holes or mesh screens) filled with test organisms in the field to experience in situ conditions for a set amount of time (typically several days to weeks). Upon removal, the survival, growth, reproduction, behavior or other attributes of the organisms are determined, or contaminant bioaccumulation can be measured on surviving organisms. Burton et al. (2005) noted benefits of such tests include “more realistic exposure conditions while allowing for experimental testing to compartmentalize stressor sources and types” and a reduction in “the level of uncertainty associated with laboratory to field extrapolations.” This approach would be especially relevant for contaminant mixtures and contaminants without guidelines.

A variety of in situ cage design and deployment orientations have been used to target shallow sediments, sediment porewater or the sediment interface, which could potentially include impacts from groundwater contamination. These consist of cylinders with mesh screens on the ends (e.g., Sasson-Brickson and Burton 1991, including upstream and downstream directed tubes to allow stream water circulation within the chamber) or sides (e.g., Burton et al. 2005) or both (Burton et al. 2012; or with bottom end open for filling with site sediment; e.g., Soares et al. 2005), which could be placed i) on the sediment with one screen down to assess exposure at the sediment interface; ii) partially buried in sediment (including screens) to assess contaminated sediments; or iii) completely buried in sediment for exposure to porewater contaminants (Burton et al. 2005). These could be deployed singly or in groups, such as the Sediment Ecotoxicity Assessment (SEA) Ring (Burton et al. 2012), with a variety of types of vertically oriented exposure cages, or in situ cages placed in a larger box-like container that had the bottom covered with grated stainless steel, thus allowing depositional sediment to contact the organism-filled cages (Cervi et al. 2020). These studies, as for other sediment-focused in situ caging studies (e.g., den Besten et al. 2003), have used a variety of different benthic organisms, with amphipods (e.g., *Hyalella*) and various worms and insect larvae (e.g., *Lumbriculus, Chironomus*) being quite common. Reported applications of in situ toxicity cages to assess benthic zone impacts from contaminated water entering from the subsurface have been limited. Most focused on contaminated sediments as the source. Sasson-Brickson and Burton (1991) and Cervi et al. (2020) focused on contaminant transfer from sediments via diffusion, presumably, given that there was no mention of groundwater flow. In contrast, groundwater flow was a critical factor in the study by Greenberg et al. (2002), which investigated the potential toxicity of sediment pore water associated with river sediments contaminated primarily by chlorobenzenes. They noted reduced survival of several invertebrate species for chambers half-buried in the sediment and under upwelling conditions (either groundwater discharge or hyporheic return flow), whereas downwelling conditions were associated with reductions in both toxicity and bioaccumulation of chlorobenzenes. Only one published study has applied in situ toxicity testing to what would traditionally be considered a contaminated groundwater plume (i.e., a concentrated zone of contaminants derived from a source more distant than the surface water sediments) discharging to a surface water body. With this type of exposure, the magnitude and direction of groundwater flow and its interactions with surface waters will likely play a more critical role. Rosen et al. (2012) tested the application of the SEA Rings at an estuary potentially adversely affected by groundwater seepage from a nearby landfill site. They assessed survival and contaminant bioaccumulation on several species and post-exposure feeding rate for polychaetes. However, they measured only low levels of contamination (e.g., reaching only trace concentrations of naphthalene and 1,2,4-trichlorobenzene) in porewaters associated with the groundwater discharge at their testing locations, as the main plume was missed. This likely attributed for the observed lack of adverse toxicity responses. To the best of our knowledge, there are no studies in the scientific literature that report on in situ toxicity caging where highly contaminated groundwater (a plume; non-sediment source) is discharging to a surface water body.

Here we share our experiences investigating the application of in situ toxicity caging tests to target groundwater contaminant discharge zones within receiving surface water bodies. The objective of this work was to assess and potentially improve the caging test equipment and its application to groundwater-sourced contamination, building upon the method development performed for those few studies targeting benthic exposure from subsurface water contamination noted above. Application for impacts to groundwater ecosystems (stygofauna) is not considered here. Four sets of tests were performed from 2010 to 2022 (Table 1) at one of three previously reported sites. These include one river site affected by an overlapping pair of volatile organic contaminant plumes (HRM site (Fig. S1); Roy et al. 2018) and two historic landfill sites with plumes discharging to a pond (HB site (Fig. S2); Hua et al. 2023) and a small urban stream (DC site (Fig. S3); Propp et al. 2022). Past measurements at each site revealed many contaminants in discharging groundwater at concentrations above their WQG for aquatic life at some locations and times, though not all contaminants found have guidelines.

**Table 1:** Summary of the in situ toxicity tests and their conditions

Study	Approach	Cage design	Cage orientation (no. cages)	Cage locations	Test organism (no. / cage)	Day # of counting
HRM 2010	Reference	Standard	H (8); HB (6); V (8)	1 E & 1 W	*Hyalella* (10)	2, 4, 7; 3, 6 for HB
HB 2019	Reference	Standard	H (15); V (15)	15 across pond	*Hyalella* (15)	7, 14, 21, 28
DC 2019 Stretch B Stretch C	Gradient	Standard			*Hyalella* (15)	7, 14, 21, 28
			H (5; S only); V (10)	5 N & 5 S		
			H (5; N only); V (10)	5 N & 5 S		
DC 2022 Test 1 Test 2		Hybrid	N/A^ξ^ (16; 8 × 2 organisms) (32; 16 × 2 organisms)		*Hyalella* (10), *Chironomus* (10)	5
	Reference			2 N & 2 S		
	Gradient			4 N & 4 S		

The Reference approach considers cages as either within or out of the contaminant discharge footprint (ignoring internal concentration variation), while the Gradient approach considers measured groundwater contaminant concentrations at each cage location (placed within and out of the contaminant discharge footprint). Standard cages were deployed with different orientations (H—horizontal; HB—horizontal half-buried; V—vertical)

^ξ^N/A—not applicable as hybrid cage design has only one orientation

While it is advisable to combine in situ toxicity tests with other assessment methods in a weight-of-evidence approach for site risk assessment (Burton et al. 2002), the focus here is on demonstrating and evaluating the method more than assessing the risks at each site. The study examined both a standard cage design (cylinder with mesh on both ends) with various in situ cage orientations and a hybrid cage design (mesh on ends and one side), which may better represent benthic conditions in a stream with groundwater discharge; all are described further below. Fully buried cages, as described by Burton et al. (2005), were not considered given the preponderant low dissolved oxygen (DO) conditions of the groundwater at these sites. The study also considered two types of organisms and assessed two toxicity endpoints: organism survival, applied over different time intervals, and, for some tests, mass of survivors at the end of the test period, representing organism growth. It also demonstrates two field approaches to cage placement for exposure evaluation that target groundwater-sourced contaminants: a Gradient approach and a Reference approach (described in detail below). Importantly, for both approaches the caging locations were restricted to a single site (i.e., as opposed to using a distant “clean” reference site) to minimize differences in overall groundwater conditions, aside from the target contamination. Finally, potential influences of site groundwater and surface water conditions are also discussed. Findings for the groundwater chemistry, but for tests carried out in 2022, and the groundwater discharging conditions have previously been published for all sites (Roy and Bickerton 2010; Roy et al. 2018; Hua et al. 2023; Propp et al. 2022). Both sets of information guided the choice of caging locations within and around the contaminant plume discharge areas.

## Methods

### Caging Test Approach

The caging approach refers to how the in situ cages are located and what contaminant data are used to distinguish the impact from the contaminant. Here either a Gradient approach or a Reference approach was used, but with all caging locations placed at a single site, as noted above. For the Gradient approach, caging locations were chosen to cover various locations within the plume, at the edge of the plume and outside of the plume, to span a range of contaminant concentrations from very high (heart of plume) to very low or negligible (background). Actual contaminant concentrations at the caging locations, determined from co-located samples of shallow sediment porewater, were used in the assessment. This sampled porewater is typically discharging groundwater at these sites, but it could possibly be downwelling surface water at a few locations. For the Reference approach, cages were placed either within or outside of the delineated contaminant plume footprint (avoiding the edge of the plume), based on its general delineation by recent contaminant sampling or other methods. Thus, caging locations were categorized as either ‘contaminated’ (in-plume) or ‘background’ (reference), respectively, with no consideration for variation in contaminant concentrations within a plume for the assessment. For the sites in this study, the reference or background locations were on the opposite side of the stream or pond from the landfill.

### Groundwater Sampling

The methods of groundwater and surface water sampling, sample handling and chemical analyses (major ions, alkalinity, ammonium, metals, volatile organic compounds and several other potential contaminants (full list in Table S2); performed in Environment and Climate Change Canada laboratories) for the HRM, HB and DC sites are described by Roy et al. (2018), Hua et al. (2023) and Propp et al. (2022), respectively. Shallow groundwater sampling within the surface water sediments was performed using either a drive-point profiler or shallow piezometers, with purging until field-measured parameters (temperature, DO, pH, electrical conductivity (EC); hand-held YSI probe) stabilized; surface water sampling consisted of grab samples. Details on sample handling and analyses are provided in the Online Resource Section B.

### Study Sites: Groundwater Contaminant Conditions and Caging Approach

The first site (HRM) is on a reach of an urban river (∼10 m wide, < 3 m deep) in the Halifax Regional Municipality, Nova Scotia (Fig. S1), that receives discharge of an overlapping set of groundwater contaminant plumes, one of petroleum hydrocarbons and one of chlorinated solvents, from two distinct but proximate point sources from the east side of the river (Roy and Bickerton 2010, 2012; Roy et al. 2018). For the in situ toxicity test performed in 2010, cages were placed in a small area near the east bank within the contaminant discharge footprint, while a reference set was placed near the opposite (west) bank, outside of the main contaminant discharge footprint (Table 1; Fig. S5) and in a location with a similar sediment substrate. Shallow groundwater sampling revealed plume VOC concentrations averaging 540 µg/L for combined chlorinated ethylenes (PCE, TCE, DCE, VC) and 130 µg/L for petroleum components benzene, ethyl-benzene and xylenes (BEX) for the east-side location, with many of these compounds surpassing their Canadian WQG for Protection of Aquatic Life (CCME, 2023). Only low concentrations of these same contaminant groups (30 and 2 µg/L, respectively) were measured at the west-side location (Roy et al. 2018). A potential complicating factor is impacts from groundwater conditions unrelated to the plume VOCs, such as lower DO levels or other contaminants. For this test, the west (reference) location showed similar to slightly lower DO (though both < 2 mg/L), higher iron and ammonium concentrations, but otherwise similar general chemistry (pH, electrical conductivity (EC)) and metals concentrations (Al, As, Cu, Cd, Pb, Zn) as the east (plume) location (Roy et al. 2018). The Cl concentrations (possibly from road salt) and alkalinity were slightly higher on the plume side. There is also some potential for impacts from unmeasured contaminants (e.g., pharmaceuticals, as artificial sweetener concentrations were more elevated on the east side). All this considered, any greater toxicity observed for the east-side cages is likely due to the plume constituents.

The landfill at the 40-hectare rural HB site operated from 1970 to 1986 and comprises a ~ 480-m-long (N-S) by ~ 280 m wide mound. As detailed by Hua et al. (2023), the groundwater plume emanating from this landfill travels through the surficial sand-gravel aquifer and discharges to the northern portion of a 200-m (N-S) by 80-m engineered pond situated ~ 40 m to the west (Fig. S2). The pond has a maximum depth of ~ 1.2 m, with the bottom covered by fine black sediment up to ~ 0.3 m in thickness. The pond receives groundwater discharge from the east, north, and west year-round, but may lose water to the ground in the south portion (far from the plume discharge location). Shallow groundwater sampling (2019, 2022), supported by electrical geophysical measurements (2022) and observations of shoreline seeps (Fig. S13), indicates that this long-standing plume discharges directly along the east shore, extending up to 20–25 m across the pond bottom. The discharging plume contains common legacy contaminants (e.g., ammonium, metals, benzene; Table 2), with concentrations varying spatially within the plume footprint (Hua et al. 2023), and some emerging contaminants (e.g., PFAS, OPE; based on one sample analyzed by Propp et al. (2021)). The background groundwater discharging west of the plume footprint was relatively pristine (Hua et al. 2023).

**Table 2:** Maximum concentrations of select contaminants in shallow groundwater within the landfill plume footprint and outside of it (i.e., background) at the HB site (all sampling data), and from the landfill-impacted and other (Alternate) locations from the DC site (August 2019 sampling), in comparison with their long-term freshwater Canadian Water Quality Guidelines for the Protection of Aquatic Life (CCME, 2023) or (for bisphenol A and PFOS) Federal Environmental Quality Guidelines (Government of Canada 2023)

Compound	Units	HB site		DC site		Guideline
		In Plume	Background	Landfill	Alternate	
Ammonium-N	mg/L	130	2.2	206	6.9	0.5–2^χ^
Chloride	mg/L	540	48	330	470	120
Arsenic	µg/L	21	0.35	3.0	1.3	5
Boron	µg/L	4260	88	940	95	1500
Copper	µg/L	10.7	1.3	0.4	2.2	4^ξ^
Iron	µg/L	41,000	360	73,000	23,000	300
Nickel	µg/L	160	4.2	2.4	1.1	150^ξ^
Benzene	µg/L	1.6	0.18	34	0.6	370
Bisphenol A	µg/L	nm	nm	7.4	nd	3.5
PFOS	µg/L	0.19	0.002	2.6	nd	6.8

^Χ^estimated range based on site pH and temperature; ^ξ^ based on the range of hardness observed on site (240–1600 mg/L CaCO_3_); nd—non-detect; nm—not measured

The DC site has three adjacent historical municipal landfills, sequentially operated from 1960 to 1963, along 0.5 km of Dyment’s Creek (< 5 m wide, typical base flow depth < 0.3 m; Fig. S3), an urban stream in Barrie, Ontario. The streambed and nearby aquifer sediments are fine sand to gravelly sand, with some fine surficial sediment deposited along the stream banks (Fitzgerald et al. 2015). The in situ caging tests, along with detailed sampling of discharging groundwater, were performed at two short reaches–—a 20-m-long straight section adjacent the middle landfill (Stretch B) and a 40-m-long meandering section adjacent the west-most landfill (Stretch C), both of which are predominantly gaining (Roy and Bickerton 2012; Propp et al. 2022). A Gradient approach was used for toxicity testing at this site, but for a preliminary test in 2022 that used the Reference approach, because shallow groundwater sampling revealed that leachate contaminants (legacy and emerging; some given in Table 2) varied substantially spatially, even though these were only impacting the side of the stream adjacent the landfill at both study reaches (Propp et al. 2022), which was the south side for Stretch B and the north side for Stretch C. Also, other urban contaminants were detected at some locations on the opposite side of the stream (see chemistry for ‘Alternate’ locations; Table 2).

Of note, measured concentrations may not exactly represent that of the groundwater discharging around or through the cages despite the proximity of the shallow groundwater sampling (within 0.3 m horizontal, within 0.3 m depth, of the cage). This is because groundwater contaminant concentrations in surface water sediments can vary substantially even at such small scales due to complex groundwater flow paths (e.g., Conant et al. 2004) and strong biogeochemical gradients (e.g., Lorah et al. 2009).

### Cage Design and Orientation

**Fig. 1:**
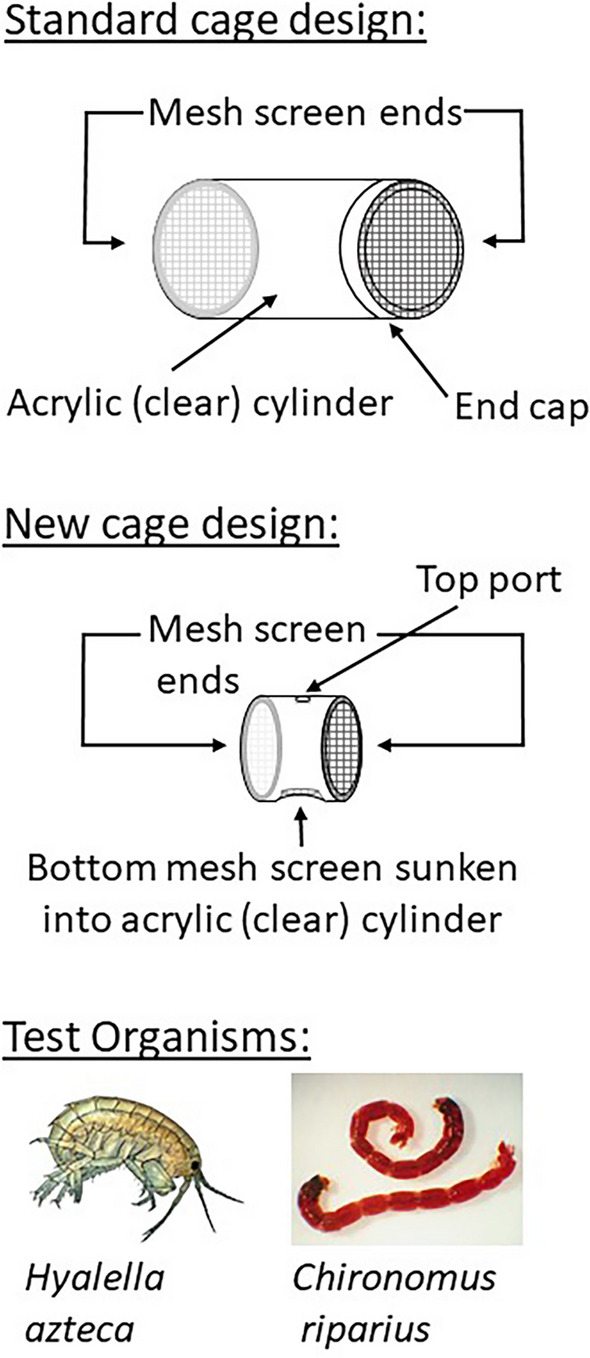
Depictions of the standard and new cage designs and the test organisms used in the various in situ toxicity tests (as summarized in Table 1)

Most of the tests used a “standard” acrylic cylinder cage (Grapentine and Rosenberg 1992), either 7.3 (HRM) or 12 (HB, DC) cm long by 7 cm in diameter (so 331 and 515 cm^3^ volumes, respectively) and consisting of two pieces fit securely together (friction fit or with rubber gasket, respectively), with 250-µm mesh plastic screen on each end (Figs. 1 and S4) to facilitate flow through the cage. Three types of cage placement (or orientation) in the field were used. The typical placement, with the cage lying horizontal on the sediment surface with mesh ends aligned with the surface water flow, if any, has been applied to surface water contaminants, but might limit exposure to discharging groundwater contaminants due to dilution in the overlying water. Groundwater-focused orientations with the standard cage included it being (i) partially buried horizontally in the sediment, but with no sediment added inside the cage as in Burton et al. (2005), and (ii) placed vertically, half-buried in the sediment. The former would potentially allow some groundwater to enter through the half-buried ends, and also limit surface water inflow closer to the sediment boundary, possibly resulting in less dilution of the discharging groundwater. The vertical orientation would only allow the flow of discharging groundwater through the cage, so long as discharge conditions (i.e., upwelling) prevailed over the study period, resulting in potentially 100% groundwater exposure. We subsequently developed and tested (in the DC 2022 tests) a novel cage design (Figs. 1 and S4), comprising a shorter (length and height) cylinder (volume 45 cm^3^) with mesh on both ends but also over an extra opening on the side of the cylinder to be placed against the sediment bed. This allowed surface water flow through the cylinder, though more restricted to that near the sediment surface, but also direct upward groundwater discharge into the cylinder via the side opening, as was the case for the cage design of Sasson-Brickson and Burton (1991). This smaller cage necessitated fewer test organisms (10 rather than 15; Table 1), but otherwise is expected to provide adequate living conditions based on experience with a variety of standard cage sizes. The cage deployment details for each test performed at the three sites are summarized in Table 1 and depicted in Figs. S5, S6 and S7.

### Cage Preparation, Deployment, and Retrieval

The organism used in all the tests was the amphipod *Hyalella azteca*, but the 2022 tests also included midge larvae, *Chironomus riparius* (Fig. 1). Both sets of organisms were obtained from cultures maintained under standard conditions at the Canada Centre for Inland Waters (CCIW; Burlington, ON; but transported by truck in large buckets to Halifax for the HRM site study) for ongoing laboratory toxicity tests. The two organisms were deployed in separate cages, though two species, including midges and amphipods, have been grouped together in one chamber previously (Burton et al. 2005).

On each day of deployment, a pool of ~ 600 4–6-week-old amphipods or 8-day-old midge larvae were assembled in a large tray. Organisms were then selected from the pool and sorted into groups of 10 or 15 individuals, depending on the test (Table 1), and held temporarily in plastic containers with culture water. Sorting occurred in random order to ensure similar size distributions between groups, with assumed similar total organism mass between all cages. Each group of organisms was then transferred to a labeled cage and randomly assigned to a field location. Approximately 15 mg of TetraMin® fish food was added to each cage. Amphipod cages also received a small (~ 2 × 2 cm) piece of gauze (see Fig. S8), while midge cages received enough coarse silica sand to cover the bottom of the cage 3–5 mm deep. Cages were closed and placed into pails containing room temperature culture water for transport to the field. Within half a day, following gradual exposure to site surface water conditions in the pails, filled cages were positioned in the sediment at the test sites. Each cage was tethered to a tent peg or rebar to anchor it on the sediment, with any trapped air bubbles removed through the mesh screens using suction with a turkey baster. Pictures of deployed cages at each site are shown in Figs. S8, S9, S10 and S11.

Organism survival was measured in the field several times during each study but for the DC 2022 study (Table 1). On these internal measurement days, cages were removed from each location and returned to the streambed, unless there were no survivors, one at a time, to limit stress. Cages were opened and organisms released within a white tray for counting (Fig. S12). For some tests, measurements of cage water EC, temperature and DO were made using hand-held probes (YSI); EC was converted to specific conductance (SC; normalized to 25 °C) using the equation in Hayashi (2004). Prior to redeployment, cage screens were gently scrubbed of any accumulated debris or algae, if necessary, and another 15 mg of amphipod food was added along with the surviving counted organisms to the cages. Cages were returned to the exact same location, though some disturbance of the sediment may have altered the conditions somewhat. Upon final retrieval, unopened cages were placed into pails of site surface water and returned to the laboratory, where the surviving organisms were counted as described above, dried for 24 h at 60 °C and weighed (aggregate per cage).

### Data Analyses

Statistical analyses of the water quality and test organism data were performed using Minitab statistical software (Mintab Inc. 2017). Most data sets were not normally distributed. Therefore, nonparametric methods were generally used to test for differences between/among experimental treatments. Analyses included median tests, Kruskal–Wallis test, Pearson and Spearman correlations, linear regression and principal components analysis (Table 3; Supporting Information, Section C.)

**Table 3:** Summary of the assessment of a potential toxic impact from contaminated groundwater (GW) discharge (from the targeted source) on survival and growth endpoints for the in situ toxicity tests, as determined by various statistical methods (Median (Med), Kruskal–Wallis (KW) or Pearson (PC) or Spearman (SpC) correlation) or general observations, distinguished by cage type and orientation (standard cages deployed with different orientations (H—horizontal; HB—horizontal half-buried; V—vertical); and hybrid cage) and test organisms (*Hyalella* (*Hy*) or *Chironomus* (*Ch*)). Full details of the targeted sources and test conditions are given in Table 1

Study	Endpoint	Caging test (Organism)	Stat. test	Tested conditions	Toxic effect?	Notes
HRM 2010	Survival	Standard H (*Hy*)	Med	E vs W	No	
		Standard HB (*Hy*)	Med	E vs W	No	
		Standard V (*Hy*)	Med	E vs W	Yes	
	Growth	Standard H (*Hy*)	Med	E vs W	No	
		Standard HB (*Hy*)	Med	E vs W	No	
		Standard V (*Hy*)	Med	E vs W	No	But with two outlier results (higher mass) for target locations
		All of the above	KW	E vs W	No	
HB 2019	Survival	Standard H (*Hy*)	Med	in vs out	No	Locations 1–9 in plume; 10–15 out of plume
			PC	cage T, DO, SC	No	
		Standard V (*Hy*)	Med	in vs out	No	2 low survival outliers; 1 linked to target source, 1 linked to low-DO GW
			PC	cage T, DO, SC	No	
	Growth	Standard H (*Hy*)	Med	in vs out	Yes	Those in plume had higher growth
			PC	cage T, DO, SC	Maybe	Positive with SC; or enhanced growth from nutrients in target source?
		Standard V (*Hy*)	Med	in vs out	Yes	Those in plume had higher growth
			PC	cage T, DO, SC	Maybe	Positive with SC; or enhanced growth from nutrients in target source?
DC 2019	Survival	Standard H (*Hy*)	None		No	Generally good survival or else non-target cause for reduced survival
		Standard V (*Hy*)	PC, SpC	GW quality^ξ^	No	Poor survival – linked to non-target contaminants or low DO in GW
DC 2022 Test 1	Survival	Hybrid (*H*)	Med	N vs S	Yes	
		Hybrid (*Ch*)	Med	N vs S	No	Mesh sealing problems likely affected the results
DC 2022 Test 2	Survival	Hybrid (*Hy*)	Med	N vs S	Yes	
		Hybrid (*Ch*)	Med	N vs S	Yes	
	Growth	Hybrid (*Hy*)	Not testable		Maybe?	Larger fewer survivors for target locations (survival of the fittest?)
		Hybrid (*Ch*)	Not testable		Maybe?	Lower mass for target locations

^ξ^Contaminant concentrations and properties (T, DO, SC) in groundwater samples

## Results and Discussion

### Study 1—HRM Site

**Fig. 2:**
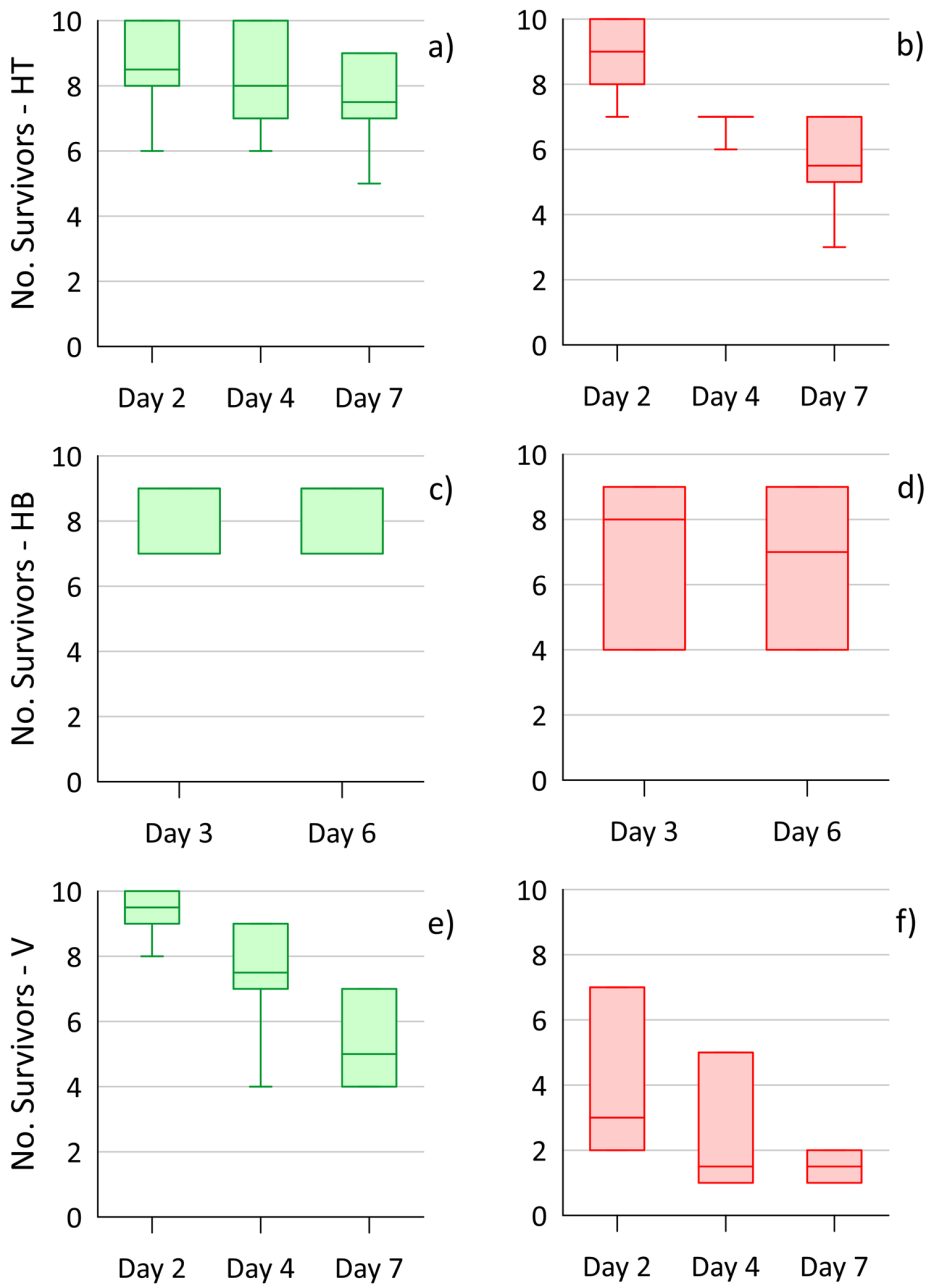
Survival results for amphipods (*Hyalella*) from 10 initial organisms/cage over the 7-day toxicity test at the HRM site, with the west location the reference area (**a**, **c**, **e**; (green)) and the east location within the plume footprint (**b**, **d**, **f** (red)) (site diagram in Fig. S5). Each box shows the data interquartile range, with middle line the median, and whiskers at maximum/minimum. The three cage orientations tested were horizontal on top of the sediment (HT; **a**, **b**; 4 cages), horizontal and half-buried (HB; **c**, **d**; 3 cages) and vertical (V; **e**, **f**; 4 cages)

The *Hyalella* survival for the 7-day 2010 caging test (Reference approach), shown in Fig. 2, was notably poorer for the vertical cages compared to the two horizontal orientations (on top of the sediment and half-buried) for each location. This greater toxic effect is not overly surprising, as water passing through the vertical cages would be largely undiluted low-DO groundwater, whereas the horizontal cages would experience substantial dilution from the well-oxygenated river water. In comparing the plume (east) to reference (west) locations, survival in both types of horizontal cage appears lower for locations within the VOC plume footprint, but the differences are not statistically significant (i.e., Median tests; Online Resource section D) considering the low cage numbers. For the vertical cages, survival declined more drastically on the east (plume) side, to < 2 survivors on day 7 for all four cages, than the west (reference). Indeed, survival was significantly different (Chi-square = 8.0; P-value = 0.005) between east and west on days 3 and 7. These results suggest that the VOC plume constituents in the discharging groundwater were causing a substantial toxic effect for the vertical cages, but possibly negligible or a minor effect for the horizontally oriented cages. However, due to the limited set of test locations and some uncertainty with unmeasured contaminants, this is not conclusive evidence.

**Fig. 3:**
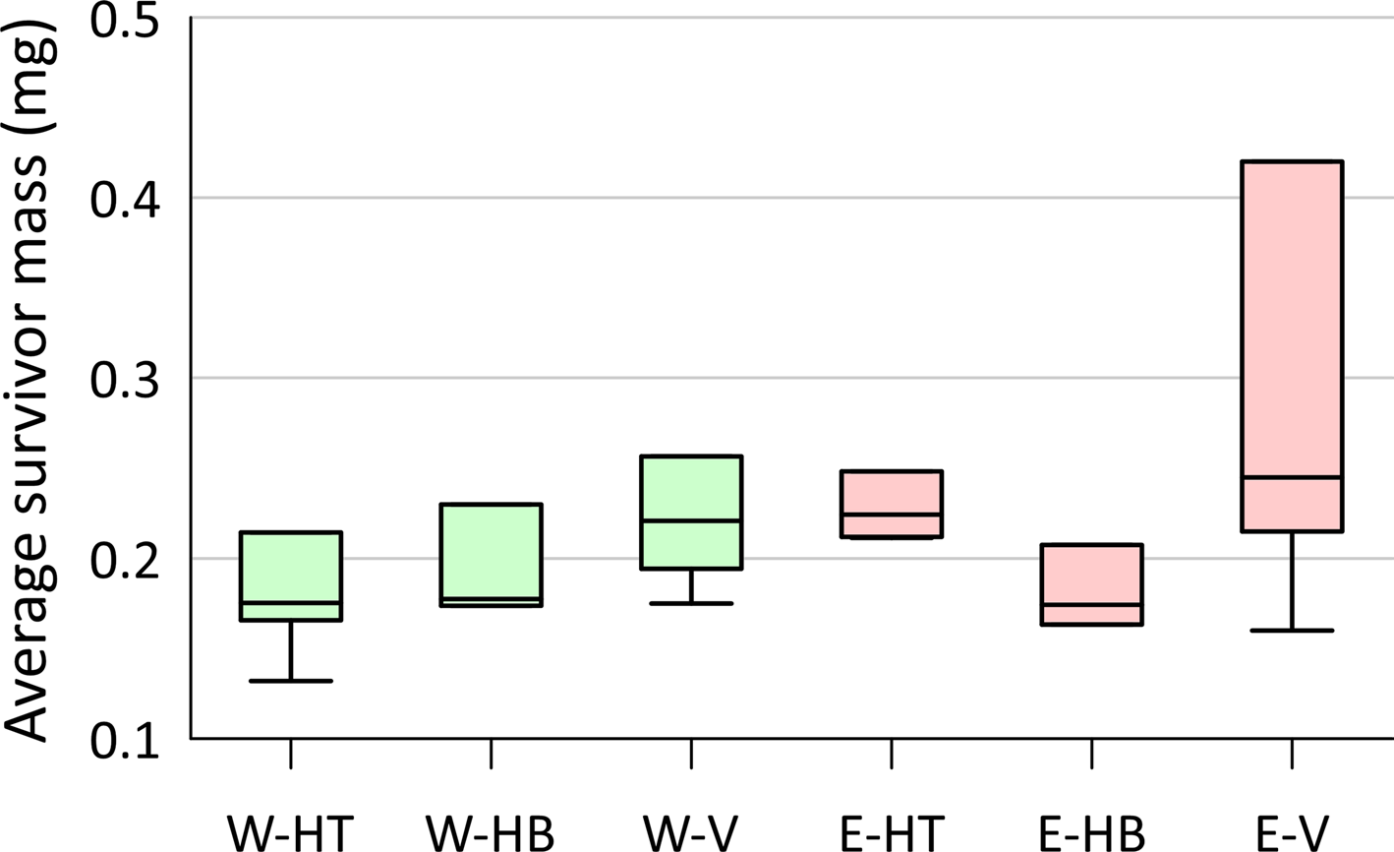
Average dry weight of surviving amphipods (*Hyalella*) collected at the end of the HRM 2010 toxicity test, comparing the west (W; outside the plume; green) and east (E; within the plume; red) caging locations (Fig. S5) and three cage orientations: horizontal on top of the sediment (HT; 4 cages), horizontal and half-buried (HB; 3 cages) and vertical (V; 4 cages)

The dry weight (mass) of the surviving organisms at the end of the test can provide an indication of potential contaminant impacts on growth, providing a secondary and sublethal measurement endpoint. This assessment assumes the random placement of organisms in the cages resulted in similar initial average total mass. For the 2010 HRM study, the average survivor mass from the different exposures (west vs. east) and cage orientations (Fig. 3) was not significantly different based on Kruskal–Wallis test (P = 0.24) and Median tests (P > 0.16; between west and east for each orientation) (see Online Resource section D for complete details). However, there were notable outliers for the vertical cages from the east (plume) side, which included the two cages with the largest dry weights. These two cages had only one or two survivors, compared to 4–9 survivors for the other five sets of cages, meaning their averages are prone to greater uncertainty. These outlier results do not suggest the plume-impeded growth of the caged organisms. Rather, it may be that some larger amphipods were better able to survive the discharging plume conditions experienced in the vertical cages on the east side.

### Study 2—HB Site

**Fig. 4:**
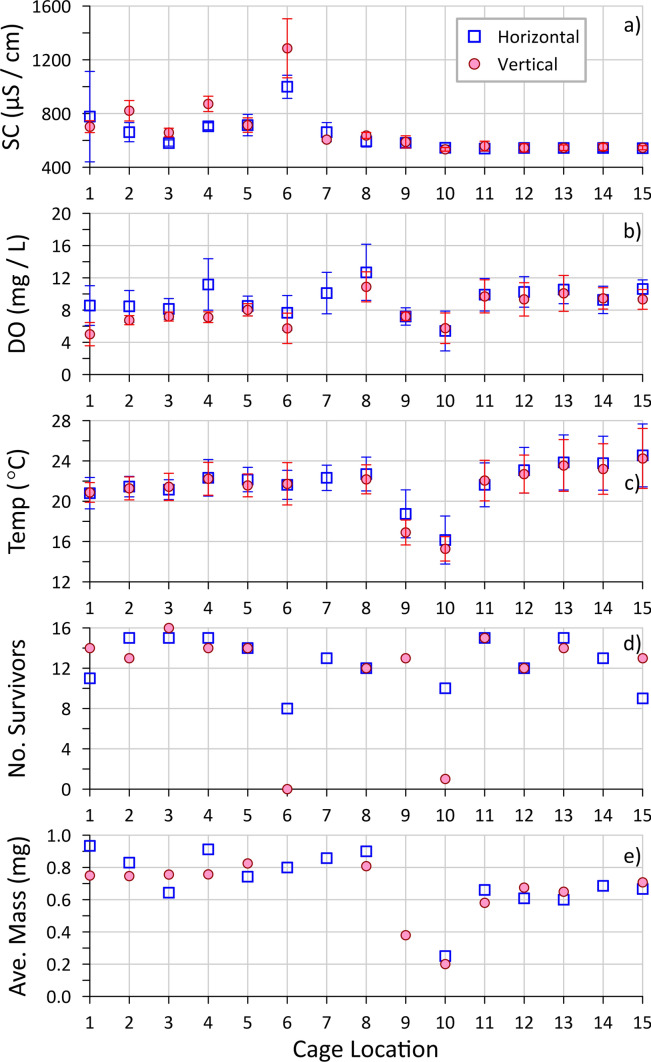
Results of the HB site in situ caging test for 15 locations (1 horizontal and 1 vertical cage at each) across the study pond (1—east (plume side); 15—west (background side)), showing averaged water quality parameters **a** specific conductivity (SC), **b** dissolved oxygen (DO) and **c** temperature, measured in the cages (with error bars representing 1 standard deviation), along with **d** minimum survival from 15 initial organisms across the four counting periods (every 7 days), and **e** average mass of survivors at the end of the study (day 28). Three cages had a malfunctioning screen, so those organism data were omitted

For the caging test at the HB site, done in 2019, fifteen cage locations were chosen within a 25-m-wide swath across the pond (east–west), just north of the main groundwater sampling transect of Hua et al. (2023) (Fig. S6), with about half within the contaminant discharge footprint (east side), as per the Reference approach (Table 1). Although exact contaminant concentrations at each cage location were not measured, field-measured SC of the in-cage water (Fig. 4a) can provide evidence of plume exposure because the shallow groundwater SC (~ 550 µS/cm in background, reaching 2000–2500 µS/cm in the plume) was strongly correlated with the contaminant concentrations (Hua et al. 2023). For both horizontal and vertical cages, the in-cage SC was consistent (540–560 µS/cm) at locations 10–15 (west side), representing the background groundwater. The remaining locations appear plume-affected (SC > 580 µS/cm, most with SC > 660 µS/cm), with locations 7–9 likely on the edge of the plume footprint. The notably higher values (e.g., location 6 with average SC > 1200 µS/cm) may reflect higher contaminant concentrations and/or fluxes (i.e., with high groundwater discharge).

The DO of the discharging groundwater was low (typically < 1 mg/L) both within and outside the plume footprint, as was groundwater temperature compared to the overlying pond water during the test (late summer) (Hua et al. 2023). Thus, lower in-cage DO and temperature (Fig. 4b, c) are suggestive of a greater groundwater flux location. Slightly lower in-cage DO and temperature were found for cages in the plume than for those in the background area, suggesting slightly higher groundwater flux coming in from the east than the west side of the pond. This likely reflects a greater hydraulic gradient from the landfill side (highest elevation). The notably lower in-cage temperatures at locations 9 and 10, which match previous groundwater and surface water observations for the pond (Hua et al. 2023), combined with slightly lower in-cage DO, indicate high groundwater flux, likely associated with an area of higher permeability in the shallow aquifer.

The in-cage SC was typically higher and the in-cage DO typically lower for the vertical versus the horizontal cage orientation in the plume footprint (Fig. 4). This indicates less dilution of discharging groundwater with pond water in the vertical cages, and therefore, greater exposure to groundwater contaminants, as was noted for the previous site (HRM). There was less difference for cages in the background locations, potentially due to lower groundwater fluxes not driving sufficient pond water out of the vertical cages (at locations 11–15) and much higher groundwater fluxes strongly impacting the conditions of the overlying water (at locations 9–10). However, none of the cages exhibited SC or DO matching that of pure groundwater, suggesting that none of the cages were fully exposed to maximum contaminant concentrations (Table 2).

A few issues impacted the assessment of the caged organism data. First, the mesh on a few cages came unsealed during the deployment, and these cages typically had substantially fewer or no survivors compared to the previous week. The data from these cages were excluded (and the seals of all cages again checked). Also, the vertical cage at location 3 had 16 organisms on day 7 counting; it is assumed that one extra organism was accidentally added initially. Finally, surprisingly, many cages held increasing numbers of organisms (i.e., 1–2 extra) in the later weeks, with some being quite small, which raises the question of whether reproduction may have been occurring or some very small native *Hyalella* somehow slipped in. As a result, the minimum number of organisms counted across the 28-day study was used for the survivor assessment. This was not deemed to have a substantial influence on the study findings but must be considered when performing long deployments of *Hyalella*.

Survival was quite high (10–15 organisms typically) for most of the cage locations, both in and outside of the plume footprint and regardless of cage orientation (Fig. 4d), with no significant difference determined by a Median test (Online Resource section D), suggesting there was little toxic impact from the discharging plume. One clear exception was location 6 with 8 survivors for the horizontal cage and 0 survivors for the vertical cage. This location had the highest in-cage SC, indicating a strong impact from the plume. It seems likely that the toxicity of the landfill contaminants was the cause of this poor survival, as other locations in the plume had otherwise similar water quality (e.g., in-cage DO that was similar or lower (location 1)). Also, as was noted at the HRM site, this result indicates a greater toxic response to groundwater in vertical cages versus horizontal cages.

There are several possible reasons for limited contaminant exposure even for the vertical cages in the plume footprint. For one, the test was performed in summer, a time when the magnitude of groundwater discharge was relatively low compared to its high in winter–spring (Hua et al. 2023). Also, the cages were placed on or into the loosely consolidated silty top sediments (Fig. S10) and these likely limited upward flow of discharging groundwater. It is suspected that groundwater flows through this top sediment largely through preferential pathways (i.e., cracks or holes facilitated by animal activity, gas bubble ebullition, high groundwater discharge, or decayed root paths; or areas of low top sediment thickness). Evidences of such preferential flow include observations of patches of murky surface water (suspended fine sediment) in sediment depressions (Fig. S14) and open holes in the ice during winter (Fig. S15). These areas are less likely to be captured within the small areas of cage deployment, and such pathways may be sealed during cage placement.

Cages at location 10 had similarly reduced survival as at location 6 (Fig. 4d), again more pronounced for the vertical cage, even though they were receiving background groundwater. However, this location is in the high groundwater discharge area (as explained above), meaning that a greater flux of groundwater with its low DO and/or other unknown toxic conditions could have elicited the observed mortality. This result demonstrates that contaminant toxicity can be obfuscated by impacts from other groundwater properties. Interestingly, location 9 was also within the high groundwater discharge area but did not show reduced survival (Fig. 4d; vertical cage only; horizontal cage data excluded because of seal failure), perhaps due to a slightly lower flux and/or slightly different water chemistry (e.g., higher DO) as suggested by the in-cage DO (> 9 mg/L).

The average mass of survivors (per cage; day 28; Fig. 4e) was greater for locations in the plume footprint (1–8) compared to background (11–15) for both orientations (Figs. S20 and S21; as determined by Median test—Online Resource section D), with a Pearson correlation showing a moderate positive relationship between mass and in-cage SC (r = 0.51, P = 0.061 for horizontal; r = 0.52, P = 0.081 for vertical; Figs. S22 and S23), respectively. This is possibly related to greater nutrient inputs from the plume (which had elevated concentrations of soluble reactive phosphorus and ammonium; Hua et al. 2023) supporting greater algal growth in the cage, which could act as an extra food supply. There was no difference in survivor mass for the horizontal cage at location 6, which was in the plume and showed slightly reduced survival, whereas the vertical cage at this location, the most highly plume-impacted cage, had no survivors to weigh. Lower average survivor mass (per cage) was observed for cages placed in the high groundwater discharge area outside of the plume footprint (locations 9 and 10). This result likely reflects limited growth at the lower temperatures at these locations (Fig. 4c; positive Pearson correlations with in-cage temperature; r = 0.47 for horizontal, r = 0.81 for vertical; Figs. S22 and S23), respectively and demonstrates a potential groundwater-related impact that could confound the assessment of the potential toxicity of discharging plume contaminants using this metric.

### Study 3—DC Site in 2019

**Fig. 5:**
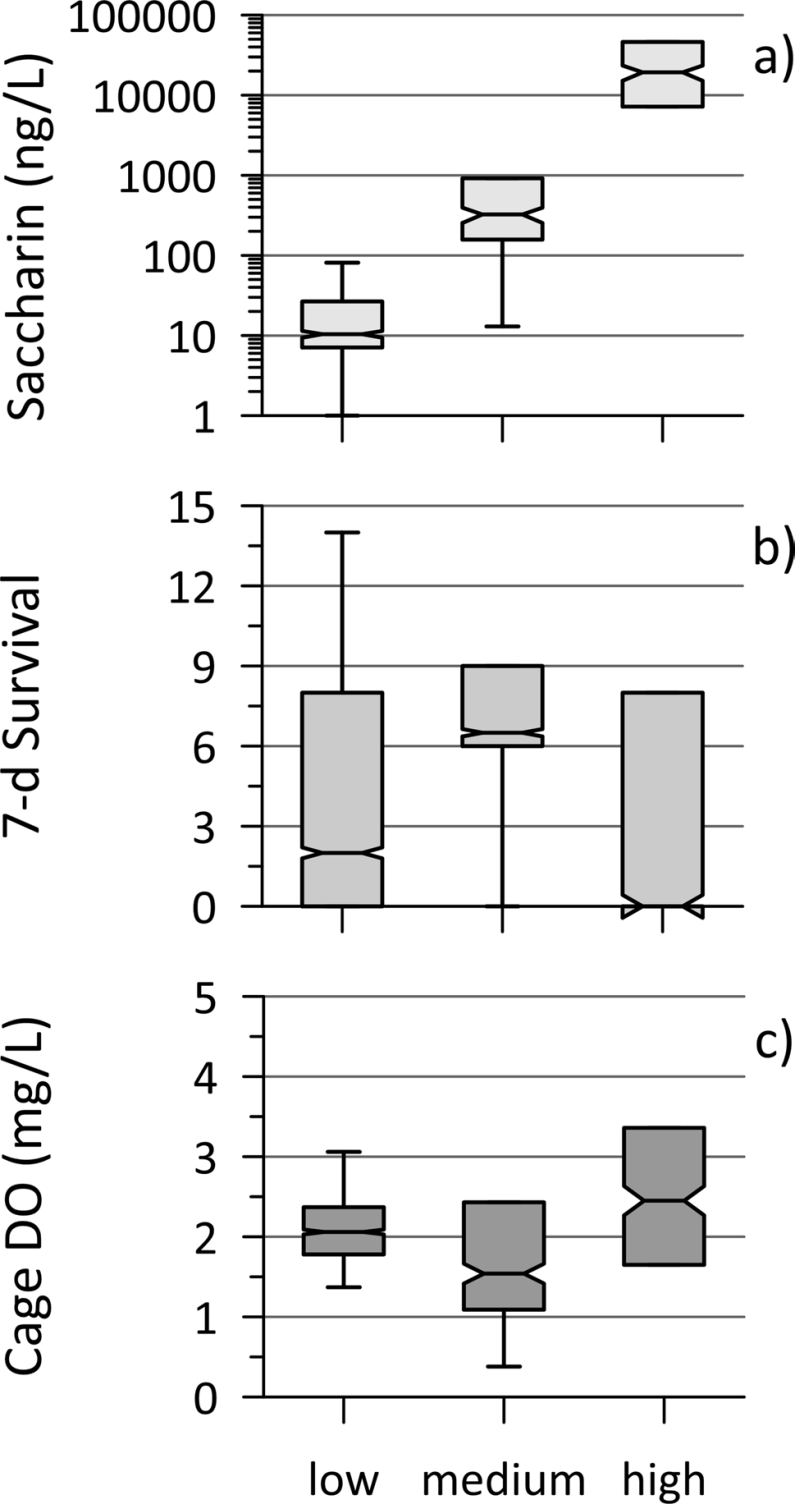
Results for the DC site (Stretches B and C combined) for 2019, including **a** saccharin concentrations in shallow discharging groundwater (August), **b** *Hyalella* survival from 15 initial organisms following 7-day exposure in vertically oriented cages and **c** dissolved oxygen (DO) measured in these same cages (day 7); for the 20 cage locations categorized as having low (13), medium (4) and high (3) levels of landfill leachate contamination

In August 2019, vertically oriented cages were deployed and co-located groundwater sampling was performed near the edge of both banks (N, S) at five longitudinal positions along both Stretch B (2, 5, 10, 15, 20 m mark) and Stretch C (0, 10, 20, 30, 40 m mark) (Fig. S7). Horizontal cages were only deployed along the landfill-impacted bank at both reaches. The caging and sampling were focused near the banks (within 0.3 m usually) because temperature-based methods revealed higher groundwater discharge rates there compared to the middle of the stream, generally (Propp et al. 2022). The twenty sample-caging locations (2 reaches × 2 sides × 5 locations) were grouped according to their levels of leachate contamination, with three categorized as high, four as medium and the remaining 13 as low. Their distinctiveness is illustrated in Fig. 5a by the concentrations of the artificial sweetener saccharin, a useful leachate tracer for historic landfills (Roy et al. 2014), which differ by orders of magnitude. This general pattern holds for many of the leachate contaminants (Table 2), though not all were perfectly correlated with saccharin, possibly due to natural source variation, potential attenuation mechanisms or the presence of different contaminant sources, including from road salt and wastewater, likely from leaky sewers. A different source is suspected at several ‘Alternate’ (i.e., not impacted by leachate) sample locations (Propp et al. 2022), as revealed by elevated concentrations of the artificial sweeteners acesulfame and sucralose (introduced in Canada in the 1990s; so not in historic landfill leachate; Roy et al. 2014). These other sources of major ions mean that in-cage SC cannot represent the level of leachate contamination here, as was applied at the HB site.

The horizontal cages at Stretch B showed minimal *Hyalella* mortality (Fig. S16), with 13 or more survivors at the end of the test for all cages but one that got buried by sediment, suggesting no meaningful toxic impact. For Stretch C, *Hyalella* survival was more variable for horizontal cages, from 14 down to 0 survivors by Day 28 (Fig. S17), though notable mortality only started occurring after two or more weeks. The highest survival was at the location with the highest saccharin and ammonium concentrations (thus, leachate impact) in groundwater, while the greatest and fastest mortality was at a location where groundwater sampling indicated no leachate impact, but only moderate wastewater impact and low chloride. These results suggest that organism survival in the Stretch C horizontal cages was not driven by groundwater leachate contamination, or perhaps by any groundwater factor, which is not surprising considering the substantial dilution expected for the near-sediment stream water flowing through these cages, as was the case at the HRM site. There was also no correlation between average DO measured in these cages and survival (R^2^ of 0.03). The causal factor(s) for mortality in the Stretch C horizontal cages could not be determined.

There were no surviving organisms in any of the vertical cages after three weeks of exposure (Figs. S16 and S17). After two weeks, there were survivors at only four vertical cage locations, including from landfill and opposite sides. This greater toxic response for the vertical cages versus the horizontal cages and its similar impact across Landfill and Alternate (non-landfill) locations (Table 2) indicate that groundwater discharge through the cages is causing mortality but that it is not strictly due to landfill contaminants. These findings also suggest that the sandy streambed here allowed for ample groundwater discharge up through the vertical cages, perhaps in contrast to the HB site, with its thick layer of fine sediment on the pond bottom. The low DO of the groundwater (< 1 mg/L), as measured at all twenty locations, likely played a contributing role in this observed mortality. Indeed, the vertical cages typically had in-cage DO of 1–3.5 mg/L.

There was a large range in survival (0–14 organisms) across the twenty vertical cages after one week of exposure, with similar variation for both stretches, which affords the best opportunity to assess if groundwater contaminants impacted survival. While the two locations with extremely high saccharin and ammonium concentrations (i.e., the highest leachate signatures; one from each study stretch), had no survivors at week 1, there was no clear pattern for survival across the three categories of landfill leachate impact (Fig. 5b). In fact, several cages in the low category and one in the medium category also had no survivors at week 1. Further, Pearson (and Spearman) correlations between 1-week *Hyalella* mortality and the landfill-associated contaminants in groundwater were typically poor (Table S3). It seems unlikely that variation in groundwater discharge was strongly affecting this exposure, given the similarity between all cages for in-cage temperature (within 0.3 °C) and in-cage DO concentrations (Fig. 5; with poor correlation with survival (Pearson r = − 0.3)). These observations suggest that exposure to leachate contaminants or to low-DO conditions was not the sole or primary driving factor for 1-week mortality in the vertical cages.

The strongest Pearson correlation with 1-week mortality was for chloride (r = − 0.51; Spearman r = − 0.59), which can be elevated in landfill leachate but is often dominated in urban areas by road salt. For Stretch B, higher chloride (and sodium) concentrations occurred at the north bank locations, opposite the landfill, and are likely from road salt wash-off from an adjacent parking lot. These locations had lower survival on average than the south bank. However, chloride does not appear to be the sole driver of the one-week toxicity variation either, as one location from Stretch C with poor survival (only 2 organisms) and the location at the B Stretch with high landfill-impact and zero survivors, had the second- and sixth-lowest chloride concentrations, respectively.

No other contaminant measured, as from different sources, gave such a strong correlation that would be suggestive of it being the sole or a primary driver of groundwater toxicity (Table S3). This suggests that multiple factors, likely including road salt supplied chloride and landfill-sourced contaminants, are leading to these 1-week toxicity results. The results may be further complicated by synergistic or antagonistic toxicity across the complex mix of contaminants occurring here. Finally, it should be noted that changes in the groundwater-surface water interactions (e.g., in response to rain events) can alter discharge fluxes and even reverse the flow direction across the streambed or change hyporheic flow paths (delineated for these stretches by Propp et al. 2022), thus potentially influencing contaminant exposure. However, such hydraulic effects were likely minimized during this test by locating cages at the edges of the stream, as these are areas of known stronger groundwater discharge (Propp et al. 2022), and by performing the test during a period dominated by base flow.

### Study 4—DC Site in 2022

The caging tests performed at the DC site in 2022 only occurred at Stretch C, again with cages at the edge of both banks (N, S), but along a more focused area (20–40 m mark) that had landfill-impacted groundwater along its full northern bank and generally lower chloride concentrations. These tests employed the new cage design and two test organisms: *Hyalella* and *Chironomus* (Fig. 1). Of note, this smaller cage design was as easy or easier to install than the standard cage, especially for placing multiple cages close together. It did not stick up into the stream as much and so seemed less likely to entangle sticks or leaves floating by, but it may be more susceptible to sedimentation. Also, the lower internal volume made measuring in-cage water properties (SC, DO, T) more challenging, though the design could be enlarged to accommodate such measurements in future.

For Test 1 (Reference approach; Table 1) in July, all cages (2 locations, 2 organisms, in duplicate = 8) against the north (landfill side) bank had no survivors after the 5-day exposure; most had many identifiable deceased test organisms remaining in the cage (2–9 bodies; including one cage with a loose seal (8 bodies). In contrast, survival was typically high (7–10 of the initial 10 organisms) for cages at the south bank, but for two *Chironomus* cages with no survivors (but no remaining dead bodies either; one of which clearly had a failed seal). This difference was determined statistically (Median test; Online Resource Section D) to be significant for *Hyalella* (P-value = 0.005) but not for *Chironomus* (P-value = 0.10), including cages with potential seal problems. This was a promising start, with the results suggesting the new smaller cage and both organisms were responsive to landfill contaminants in discharging groundwater at the north bank but not to presumably equally low DO (given past DO measurements) or other contaminants in discharging groundwater at the south bank. However, this test was for a limited number of locations and lacked concurrent measurements of groundwater contamination and surface water quality. Also, the failed cage seals were a concern, so all cages were subsequently checked and seals reinforced, if necessary.

**Fig. 6:**
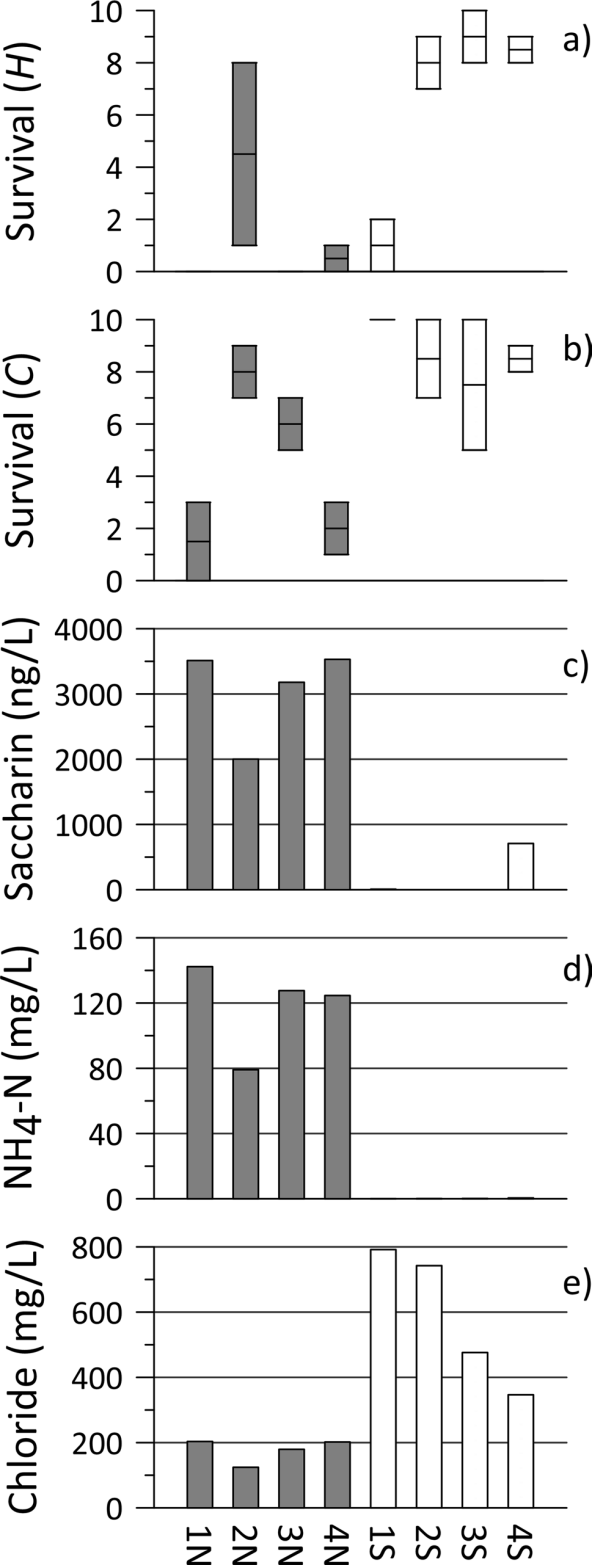
Caging and groundwater sampling results for the DC site Test 2 (Table 1), for four locations on the north (gray bars; landfill-impacted) and south (white bars) sides of the stream, showing 5-day survival for two cages each for **a** *Hyalella* and **b** *Chironomus*, from ten initial organisms, and concentrations of **c** the artificial sweetener saccharin, **d** ammonium-N and **e** chloride, in co-located discharging groundwater

Test 2 in October (Gradient approach; Table 1) deployed 32 cages (4 locations × 2 sides × 2 organisms × 2 replicates; 10 organisms per cage) in the same Stretch C segment, with the stream under predominantly base flow conditions but for a short period around deployment. Groundwater samples collected the day of cage deployment revealed that the four locations on the north bank (landfill side) were all strongly impacted by leachate contamination (see saccharin and ammonium-N plots; Fig. 6c,d). Samples from the south bank locations had much lower concentrations of leachate indicators but had higher chloride concentrations (Fig. 6e), likely from infiltration of road salt wash-off from an adjacent parking lot. There was clearly lower survival of *Hyalella* from cages on the landfill-impacted side (north; average survival of 1.3) versus from those on the south (average survival of 6.6) (Fig. 6a; Median test P-value = 0.003; further details including inter-quartile range (IQR) given in Online Resource section D). For *Chironomus* cages, the average survival was 4.4 versus 8.6 for north and south, respectively (Fig. 6b; Median test P-value = 0.012; IQR and other details in Online Resource section D), revealing a similar pattern but broadly reduced impact on survival compared to *Hyalella*. Considering only the four north-side locations, location N2 exhibited the highest survival for both species; this could be due to the notably lower leachate (e.g., saccharin and ammonium) concentrations there or it might reflect a lower groundwater flux up through at least one of those cages. For the entire data set, regression analysis (Figs. S24, S25, S26 and S27; Online Resource section D) indicated significant negative relationships between survival (both species) and saccharin and ammonium-N (all with P < 0.035). These findings suggest that the groundwater discharge of landfill contaminants was the cause of reduced survival in this test.

There was also notably low *Hyalella* survival for one set of cages from the south-side (location 1S; 0 and 2 survivors), whereas *Chironomus* had complete survival there (Fig. 6a, b). This location, beside an area receiving plowed snow from an adjacent parking lot, had high concentrations of chloride (Fig. 6e; and sodium, suggestive of road salt), though so did location 2S, with much higher *Hyalella* survival (Fig. 6a). But location 1S also had many metals (e.g., Al, Cd, Co, Cu, Cr, Ni, Pb, Sn, Ti, Zn; typical of vehicle wear and emissions) at concentrations 3 to > 10 times higher than all other south-side samples and many north-side samples too (not shown). This location was an outlier with respect to metals contamination (as shown in principal component analysis plots; Figs. S28 and S29). One location from the 2019 test, in this same area, had similar groundwater chemistry and no *Hyalella* survivors after one week. The elevated concentrations of one or multiple of these metals, possibly in association with elevated chloride, may have caused the toxicity to *Hyalella* here (and in the 2019 test), noting this organism is quite susceptible to many of these metals (Borgmann et al. 2005), whereas *Chironomus* are not as sensitive (von der Ohe and Liess 2004).

While the toxicity pattern is suggestive of an impact from groundwater discharging up from the bottom screen into the cage, there could also be an impact from stream water passing through horizontally across the side screens. This test occurred during a time of low stream flow when Stretch C was largely composed of two channels running along both banks (shallow islands in the middle). Surface water sampling revealed slightly higher concentrations of landfill contaminants in the north channel, reflecting the discharge of landfill-impacted groundwater to the northern edge of the stream (so also an effect of groundwater contaminant discharge). However, the contaminants had much diluted concentrations in the stream water and the 2019 study with standard horizontal cages showed good overall survival after one week. Further, toxicity of stream water seems unlikely to explain the low *Hyalella* survival at location 1S compared to the three other downstream cage locations on the south side. Thus, it seems unlikely the stream water quality played a role in these results. Some measure of groundwater flux at the caging locations, as by streambed temperature measurements (Conant, 2004), or groundwater contaminant flux, as through in-cage water measurements (as for the HB site), would have been advantageous.

Considering the second toxicity metric, the average mass of survivors at the end of the 5-day test showed different trends with contaminant exposure between the two organisms. *Hyalella* had larger survivor mass for north-side (average of 0.063 mg) versus south-side (average of 0.052 mg) cages, an increase of 21%, whereas *Chironomus* had the reverse (averages of 0.15 mg versus 0.20 mg, respectively; a decrease of 25%). *Hyalella* cages had few survivors on the north bank (only 10 of original 80), and its data showed a trend of larger survivor mass with fewer survivors in each cage (all cage data combined; Fig. S18), suggesting this result reflects a bias of smaller organisms dying preferentially and leaving larger organisms as survivors. Thus, any contaminant effects on *Hyalella* growth may be masked. The *Chironomus* cages had > 100 survivors (1/3 on north-side) and showed no trend for survivor mass per cage vs. survivor numbers (Fig. S19), suggesting the results do not reflect survival of the largest organisms, as for the *Hyalella*. Thus, the *Chironomus* survivor mass difference may indicate that the landfill chemicals inhibited their growth, in addition to affecting survival. Given the survivor masses were averaged per cage, and with different numbers of survivors per cage, a clear statistical comparison between the north and south sides could not be made.

## Conclusions

This presentation of the first data sets of in situ caged benthic organism toxicity tests applied to highly contaminated discharging groundwater plumes reveals promise for their use to further assess ecological impacts at contaminated sites. A summary of the findings is presented in Table 3. Increased mortality was observed at locations of high plume-contaminant concentrations at all sites, in at least one of the variety of tests, indicating a toxicity risk to benthic organisms in the three study water bodies. Results were sometimes confounded by non-target contamination (urban sites) or other groundwater conditions (e.g., high flux of low-DO groundwater), although in-cage measurements of common field parameters (temperature, SC, DO), if available given the cage and site conditions, were helpful in identifying the latter. The growth metric, determined by mass of surviving organisms, provided a less clear response to toxic conditions, with possibly competing effects of toxicity-limited growth and preferential mortality of smaller organisms.

This study also provided useful information on applicable cage deployment conditions. Animals in vertical cages were more sensitive to groundwater contaminants, but also other groundwater conditions, like low DO, than standard horizontal cages, even if half-buried in the sediment. Thus, the latter are not recommended for groundwater contaminant discharge applications. The new hybrid groundwater cage, with similarities to that of Sasson-Brickson and Burton (1991), underwent limited testing but gave a clear plume contaminant-related response. It may also reduce problems from low-DO groundwater filling the cage entirely, leaving the test organisms with no possible escape to fresher conditions in the stream water just above the sediment bed. This hybrid cage holds promise as the best design for most circumstances and deserves further testing. With respect to test organisms, the commonly deployed benthic amphipod *Hyalella* showed good background survival and strong mortality response to contaminants in all three site settings, while *Chironomus* gave a similar but more muted response in its one testing. Further testing of these and other benthic organisms to groundwater settings, particularly with low groundwater DO, is needed. A greater selection of groundwater-viable test organisms would allow for specific targeting of contaminants with select sensitive species and broader assessment of complex or new contaminants. From a practical standpoint, this study showed some challenges with groundwater-focused in situ cage testing in i) pond or wetland settings, due to thick fine sediments at the sediment interface potentially driving preferential flow paths for groundwater discharge, and ii) stream settings where sedimentation and debris could bury cages. The work also showed that an understanding of site hydrogeological and contaminant conditions, including groundwater-surface water interactions, is critical for both cage deployment and test results interpretation. In future, some measure of groundwater flux during the deployment period at the cage locations would likely prove very useful in interpreting the data.

The use of such in situ toxicity cages would be applicable to effects-based monitoring at contaminated sites, thus lessening the need for extensive expensive chemical analyses. However, ideally it would be combined with other chemical and laboratory-based measures (i.e., laboratory toxicity testing, indigenous community and habitat surveys, tissue analysis, transport and food-chain modeling, and physico-chemical characterizations; Burton et al. (2005)) to provide a weight-of-evidence approach to more confident site assessment. It would be especially beneficial for groundwater contaminated by mixtures of toxic compounds, as fits the landfill and mixed plume cases here, or by emerging contaminants without aquatic life guidelines, or by unknown contaminants.

### Supplementary Information

Below is the link to the electronic supplementary material.

Supplementary file1 (DOCX 10452 KB)

## Data Availability

Data and associated metadata are available from the corresponding author (jim.roy@ec.gc.ca) upon request.
