# Supplementary material for: Insights into In Situ Benthic Caging Tests for Ecotoxicity Assessments Targeting Discharging Groundwater Contaminant Plumes
Source: Arch Environ Contam Toxicol. 2024 Jul 3;87(1):78–93. doi: 10.1007/s00244-024-01075-9 (PMC11283422; doi:10.1007/s00244-024-01075-9)
Supplement: Supplementary file 1 — Supplementary file1 (DOCX 10452 KB) [file 244_2024_1075_MOESM1_ESM.docx]

Supplementary Information for article:

**In situ benthic caging tests for ecotoxicity assessment targeting groundwater-sourced contaminants**

J.W. Roy and L. Grapentine (Environment and Climate Change Canada)

Section A - Site Pictures:


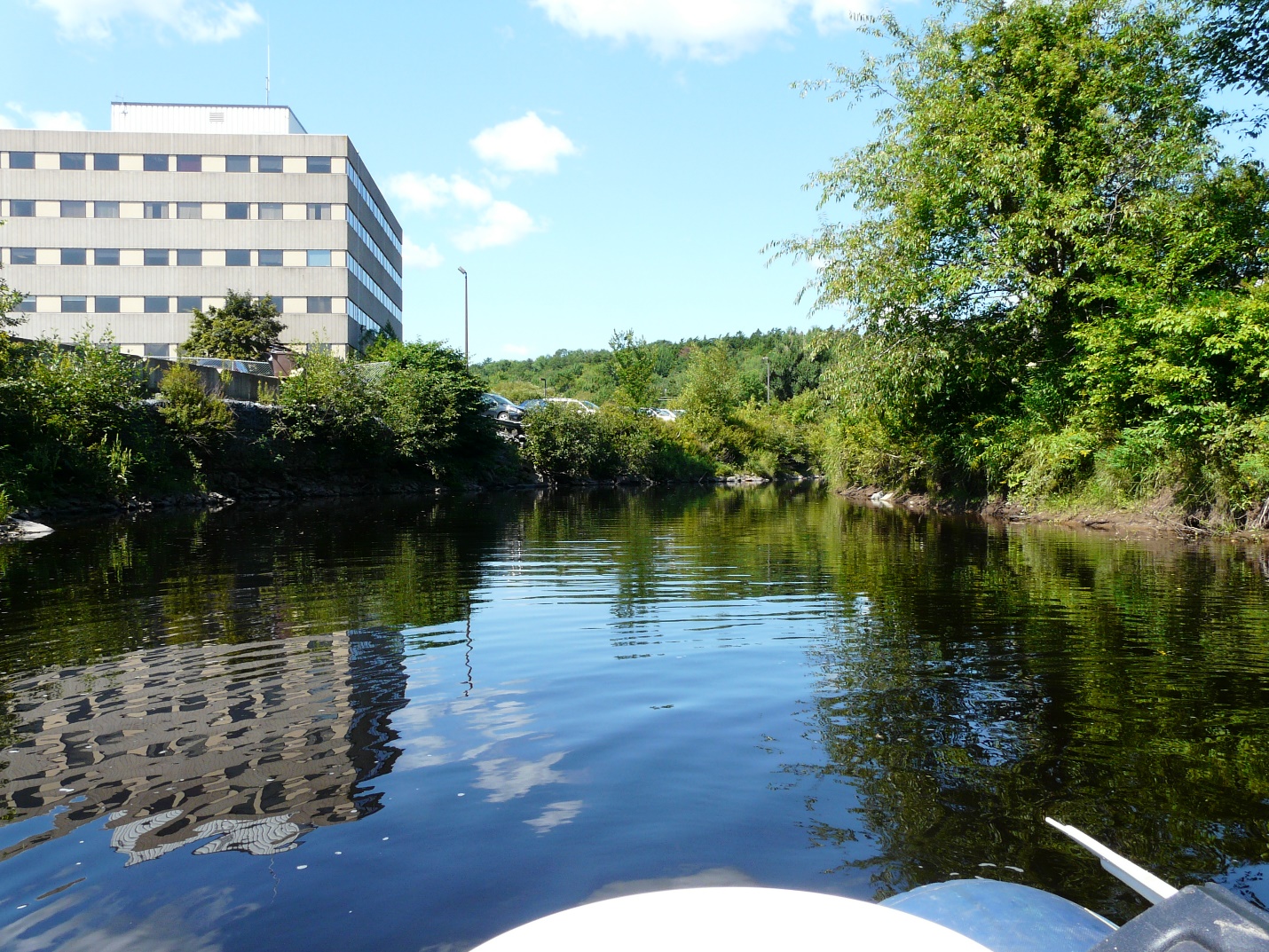


**Figure S1**. Picture of the HRM site, with the contaminant plumes coming from the east side (left).


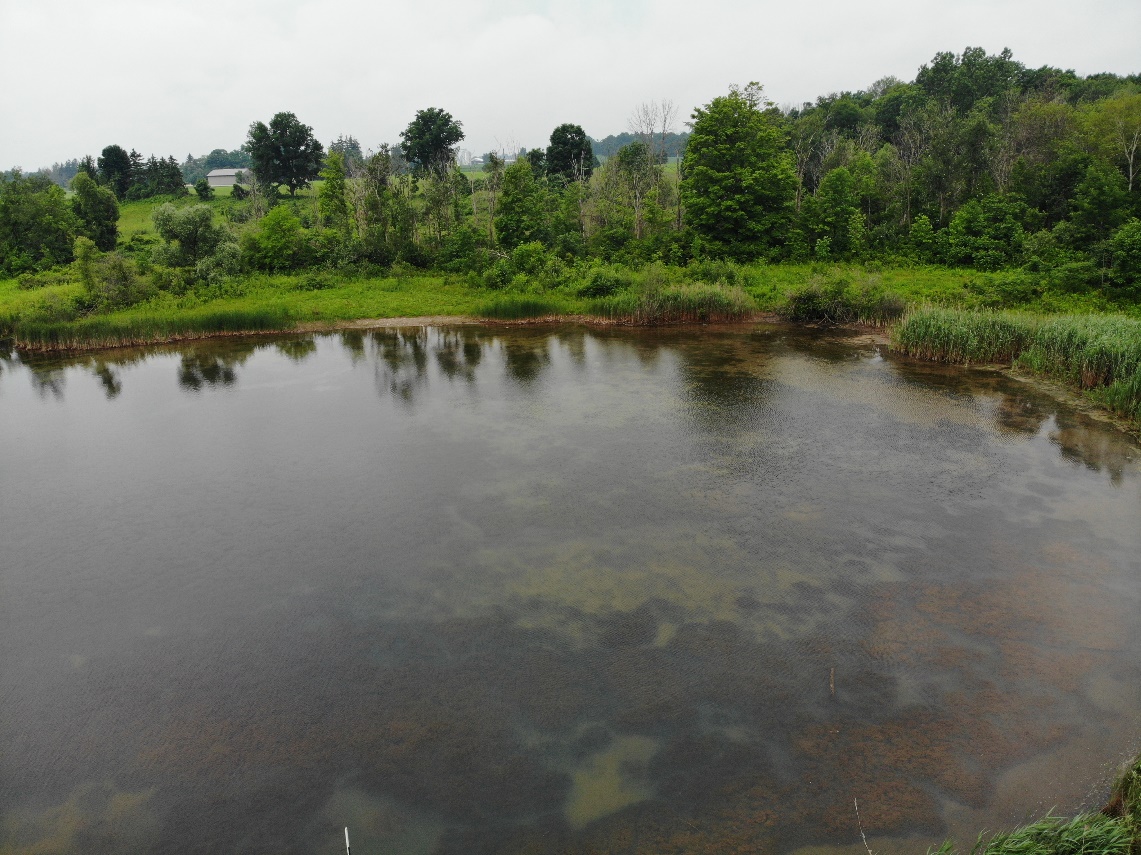


**Figure S2**. Picture of the northern half of the HB site pond (north is right).


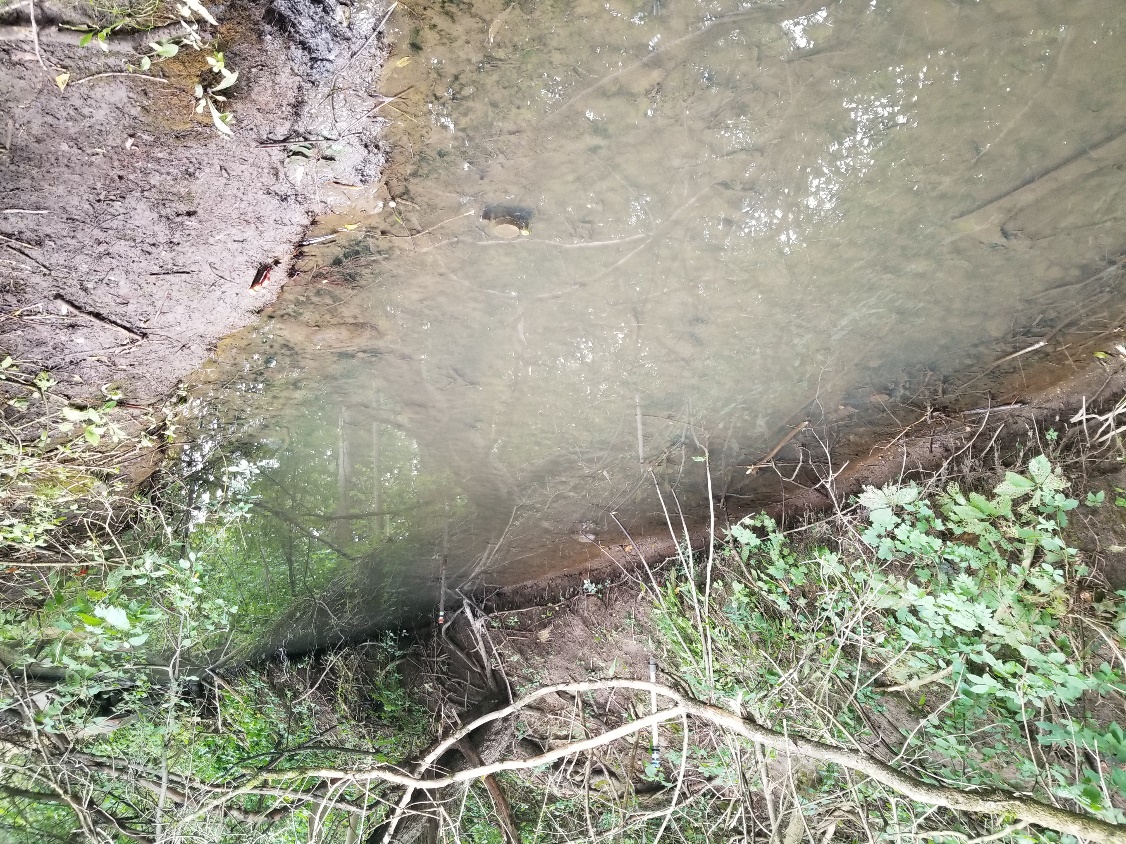


**Figure S3**. Picture of the DC stream at Stretch B (north is right).

Additional cage information:

a) b)


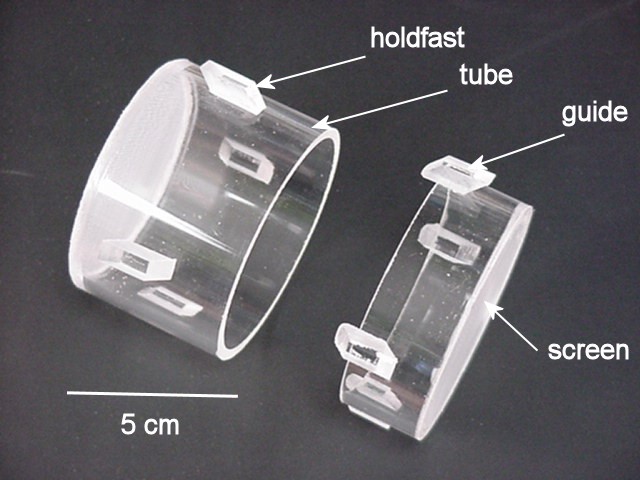

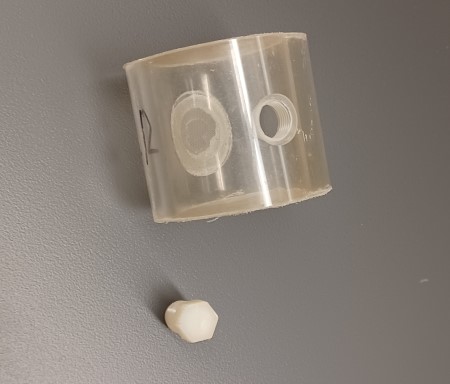


**Figure S4**. Picture of a) the standard cage disassembled, and b) the new design with plug removed.

Depictions of site caging locations:


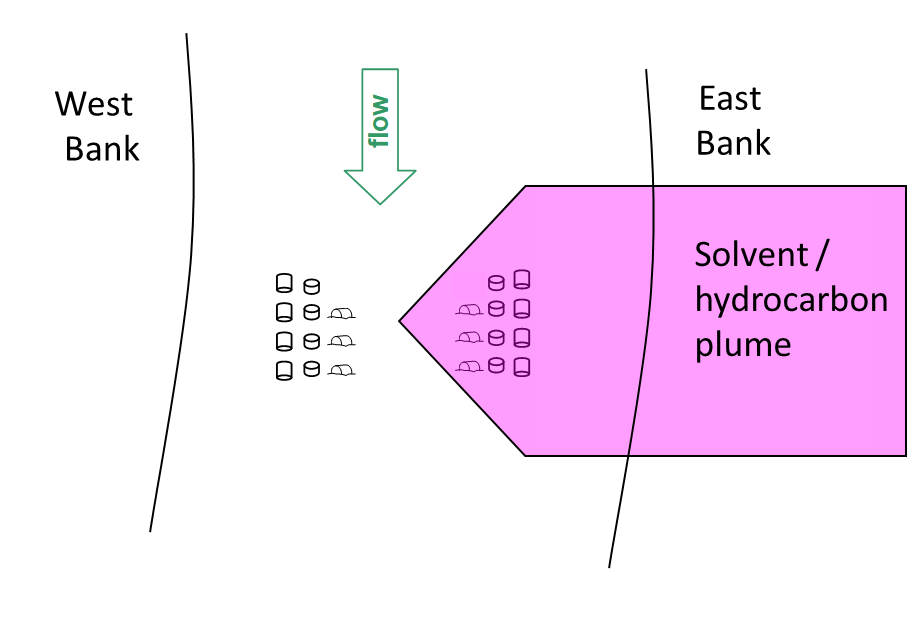


**Figure S5**. HRM site diagram showing the two caging locations (containing 4 horizontal on top, 3 horizontal half-buried, and 4 vertical cage orientations), with one in the groundwater contaminant plume footprint (east side) and one outside of it (west side); applying the Reference approach.

**Figure S6.** HB site diagram showing the 15 caging test locations, with 1-6 within the landfill plume footprint, 7-9 on the edge of it, and 10-15 outside of the footprint; applying the Reference approach. (TA is Transect E-W in Hua et al. (2023)).


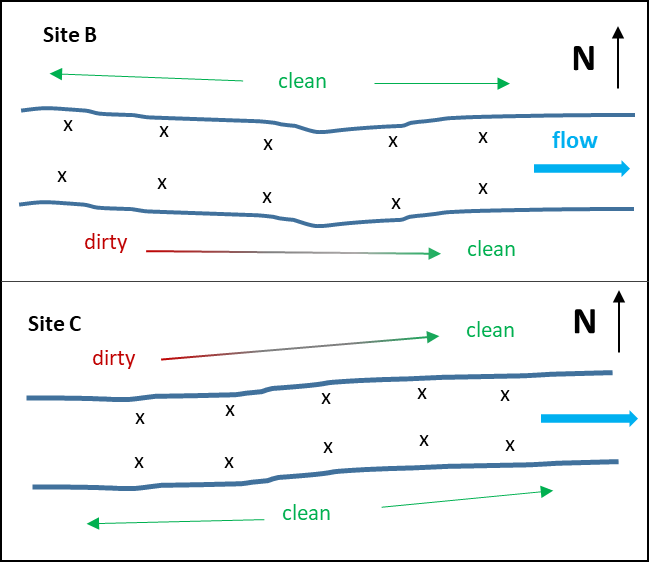


**Figure S7.** DC site diagram showing cage-sampling locations (X) for the 2019 study for Stretch (site) B (positions 2, 5, 10, 15, 20 m starting from the east) and Stretch (site) C (positions 0, 10, 20, 30, 40 m starting from the east). The 2022 study was performed at Stretch C only.

Pictures of cage deployment:


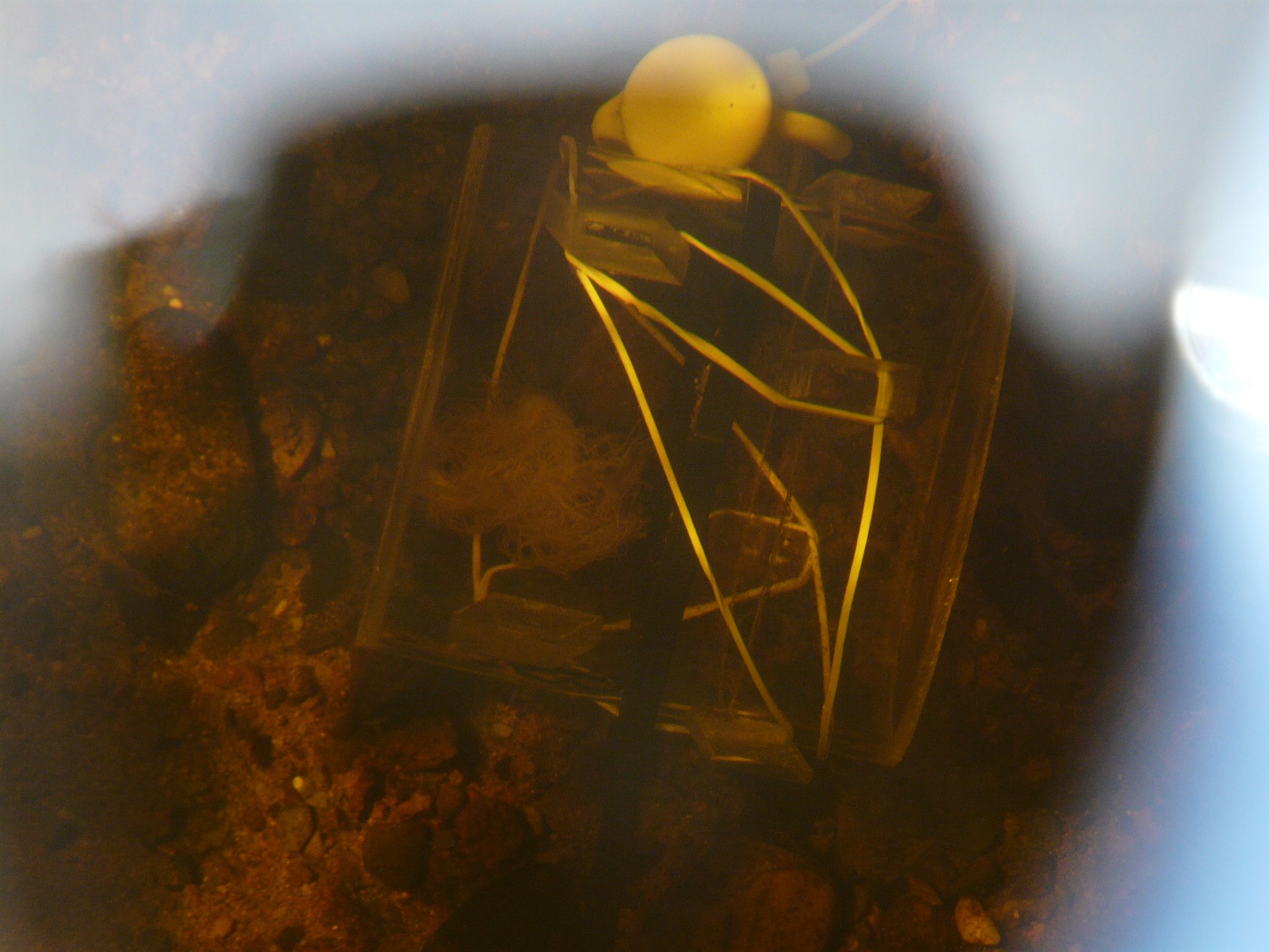


**Figure S8**. Up-close picture of a horizontal cage on the riverbed at the HRM site.


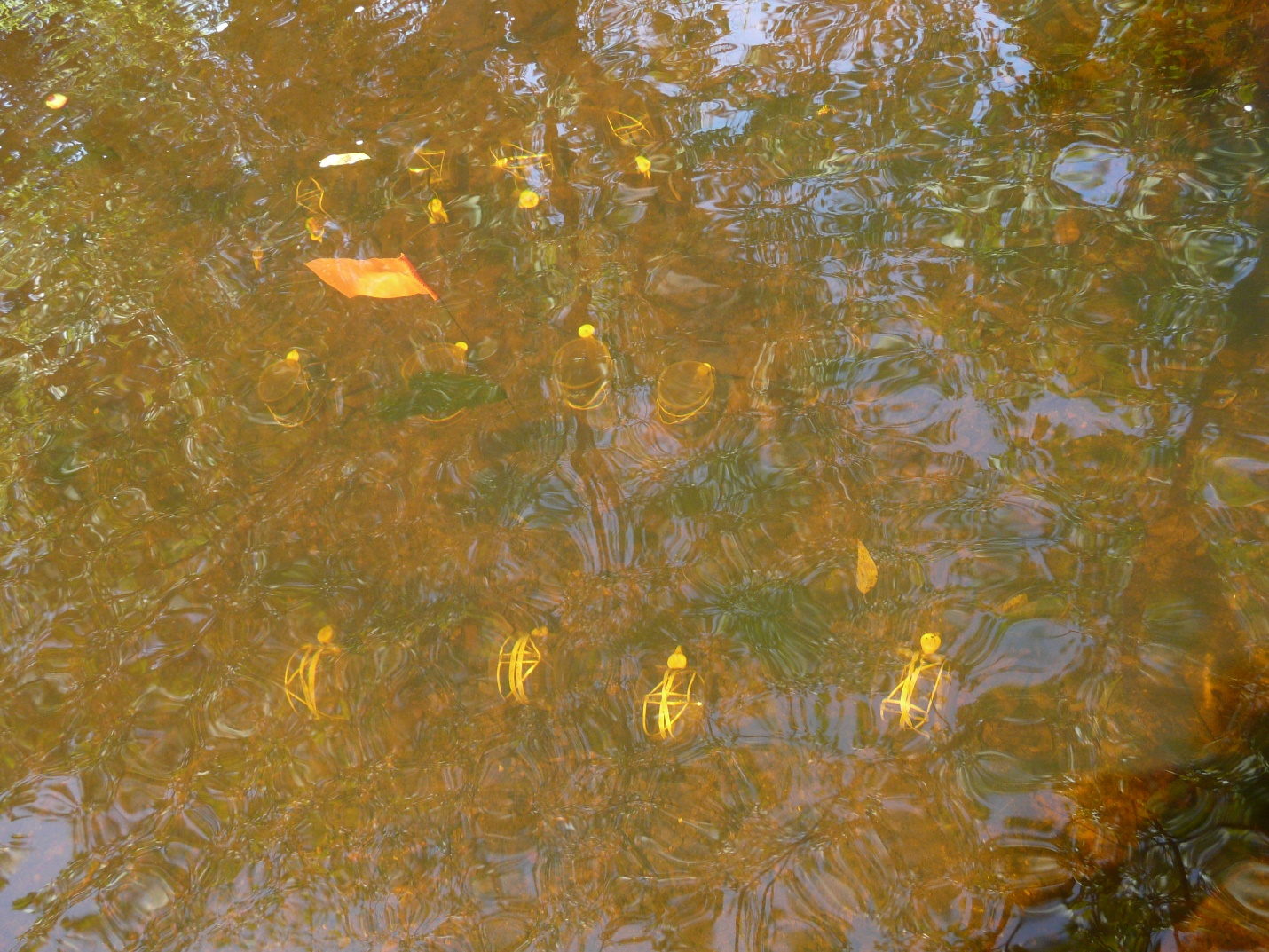


**Figure S9**. Picture of in situ cages fixed to yellow tent pegs with rubber bands in the river at one location of the HRM site (4 cages each of horizontal on top, horizontal half-buried (HB), and vertical orientations; 1 HB cage was subsequently removed from the test).


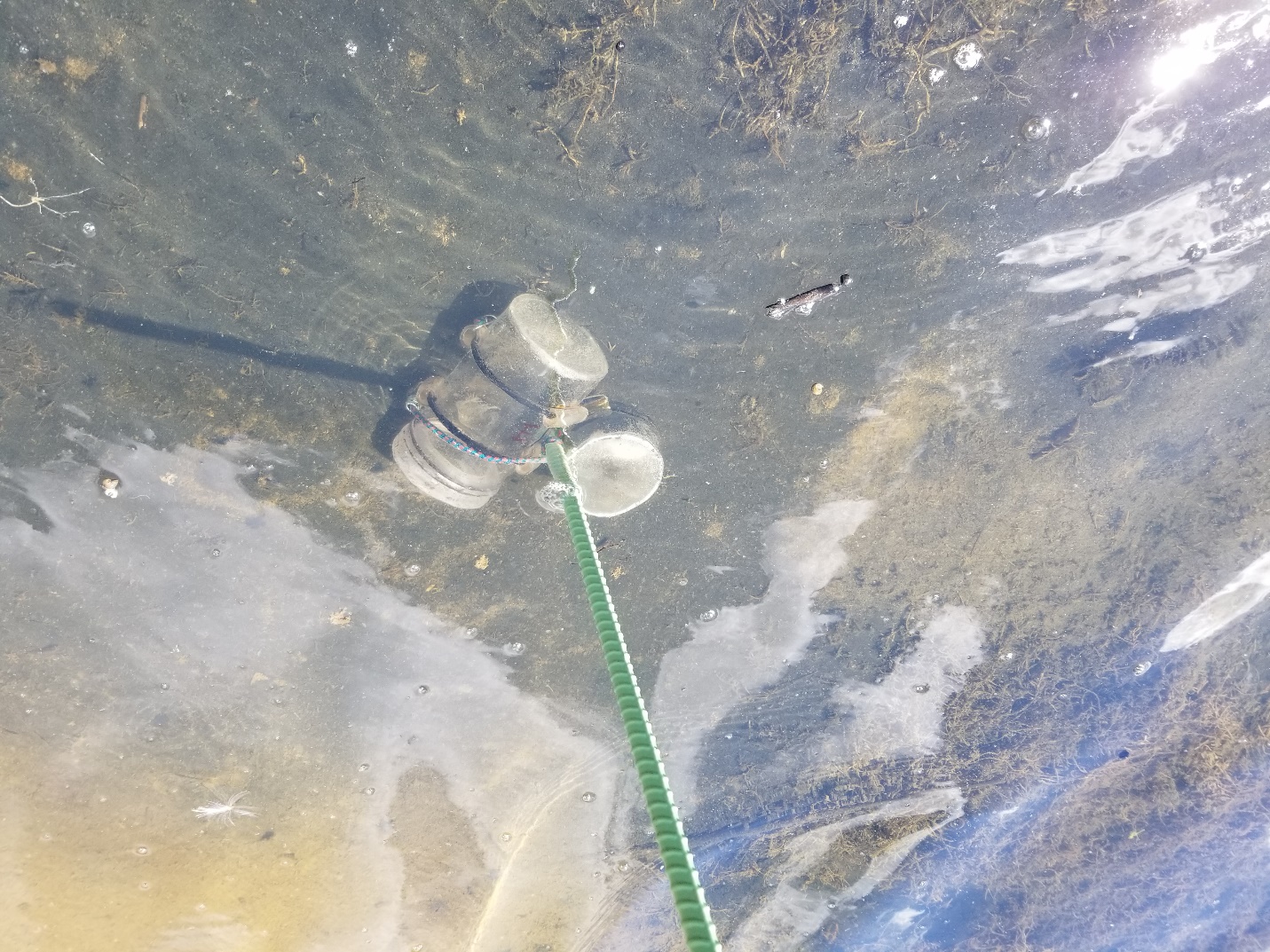


**Figure S10**. Picture of in situ cage deployment on the pond sediment at one location (1 vertical, 1 horizontal cage) at the HB site.


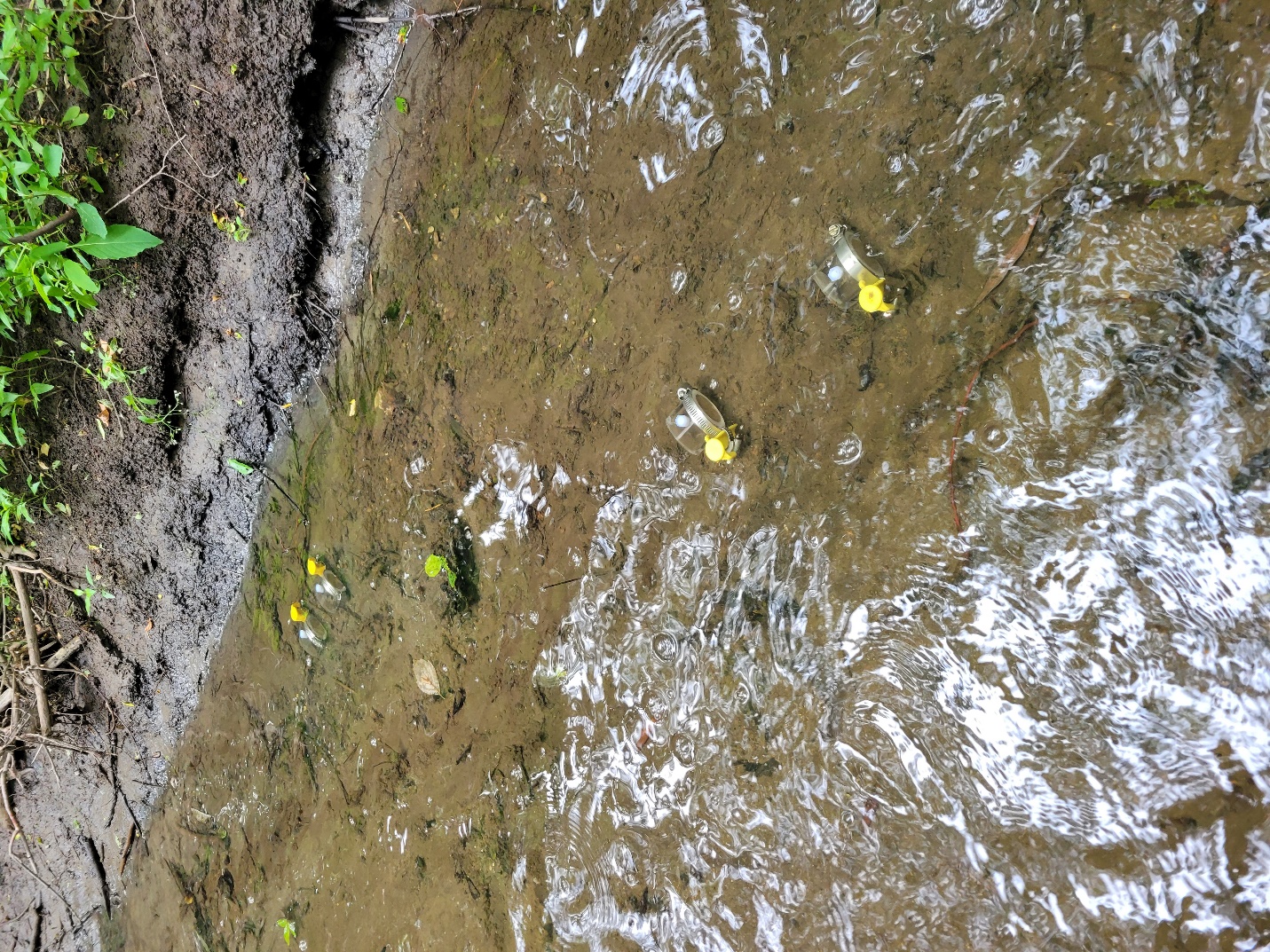


**Figure S11**. Picture of new-design cages deployed at the DC stream site (Stretch C) at two locations of the preliminary test (2022).

Additional site pictures:


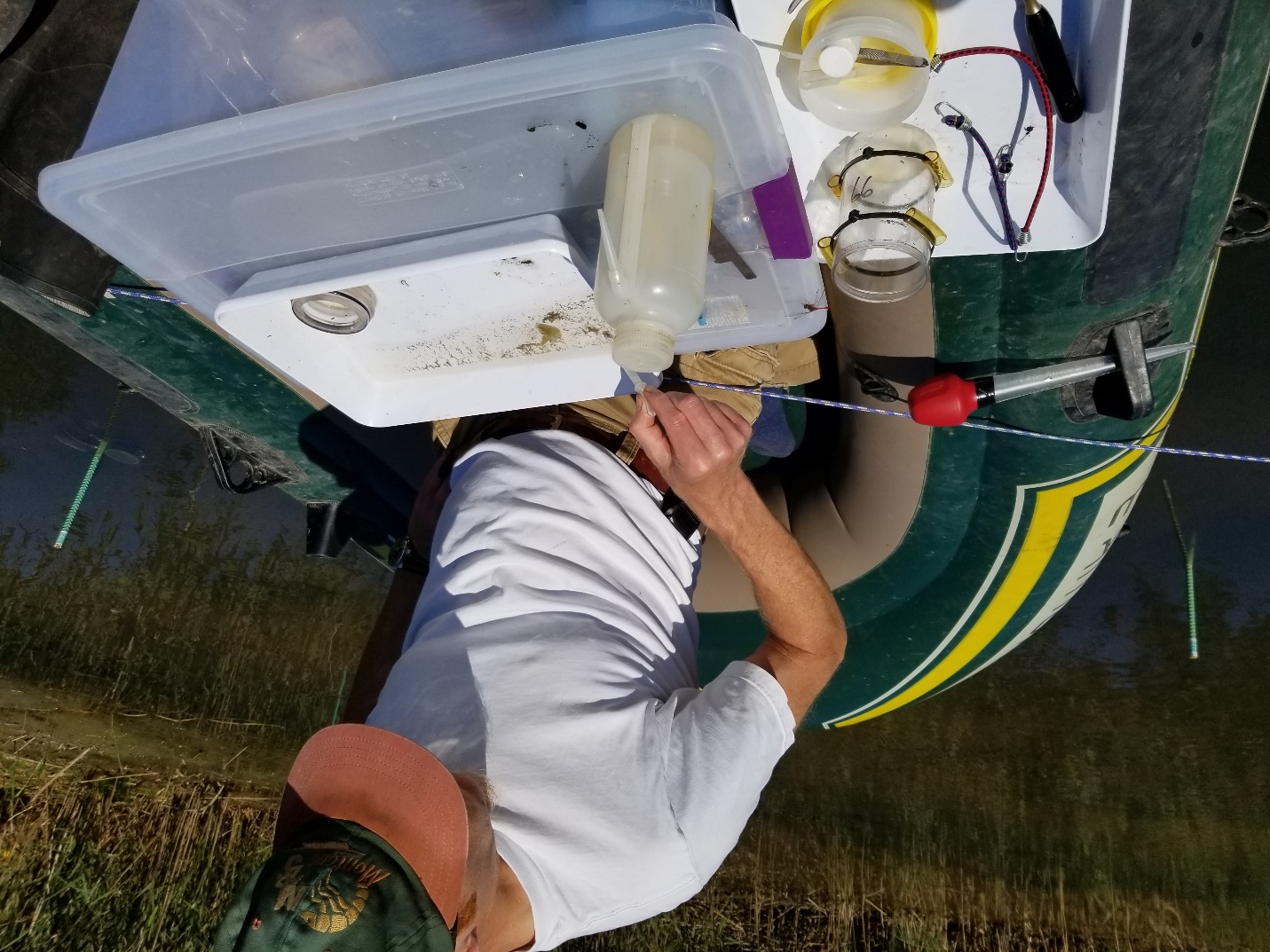


**Figure S12**. Picture of Lee counting *Hyallela* from a cage collected at the HB site.


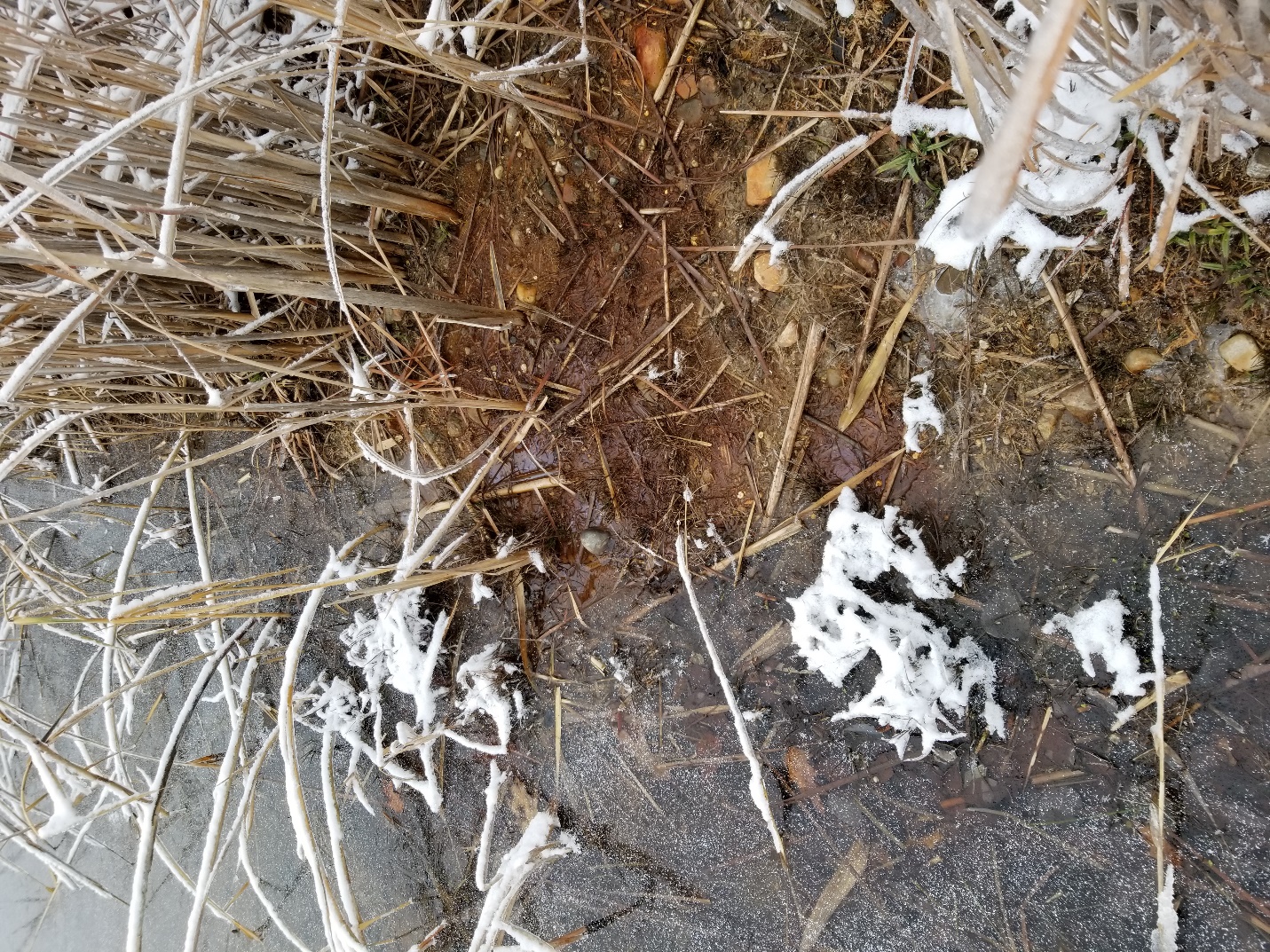


**Figure S13**. Picture of a groundwater seep area at the east edge of the HB pond, with orange-red iron staining indicating contamination from the landfill.


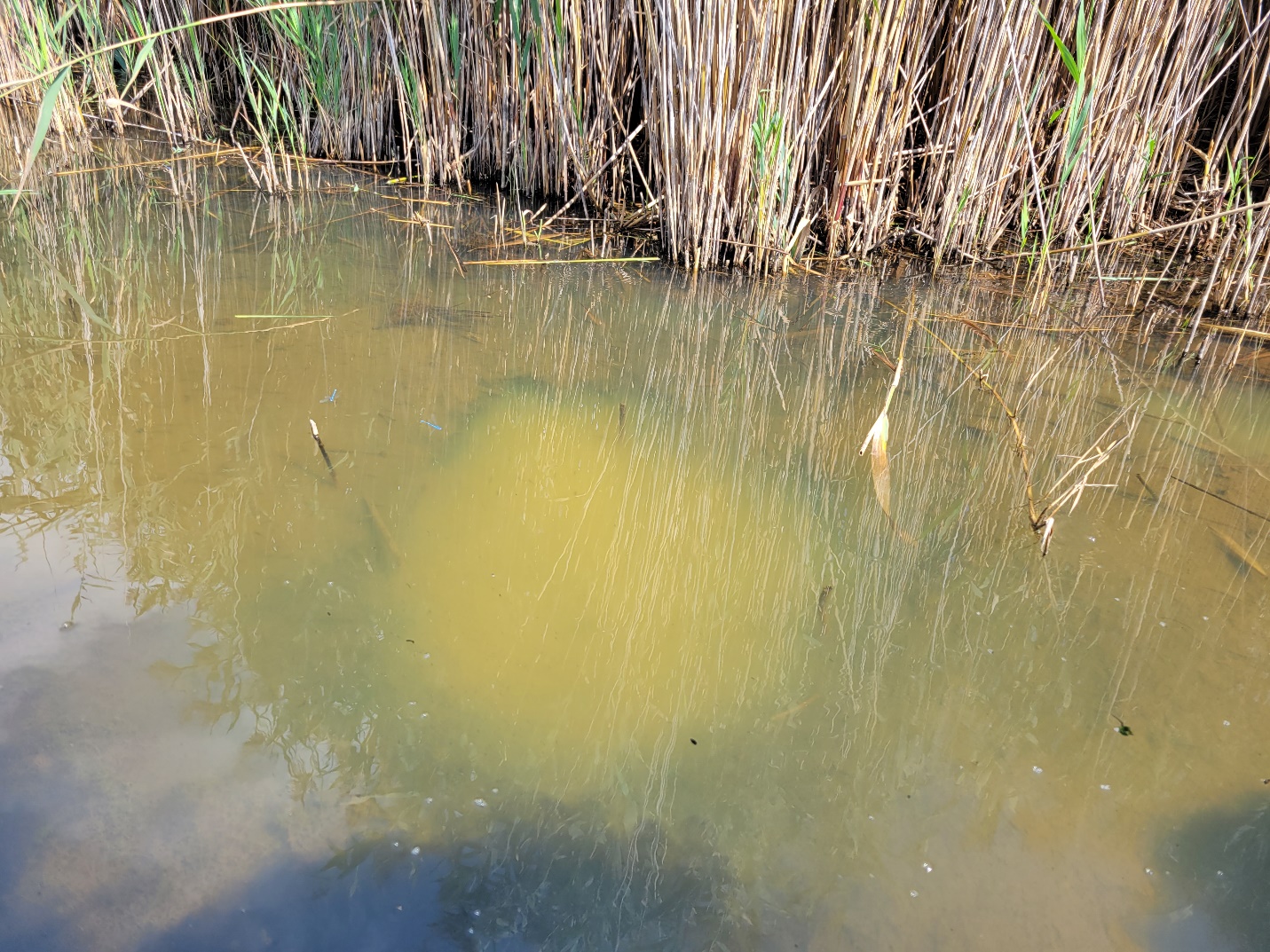


**Figure S14**. Picture showing murky water within a small depression in the top silty sediment of the HB pond, potentially a result of preferential flow in this area of thin top sediment.


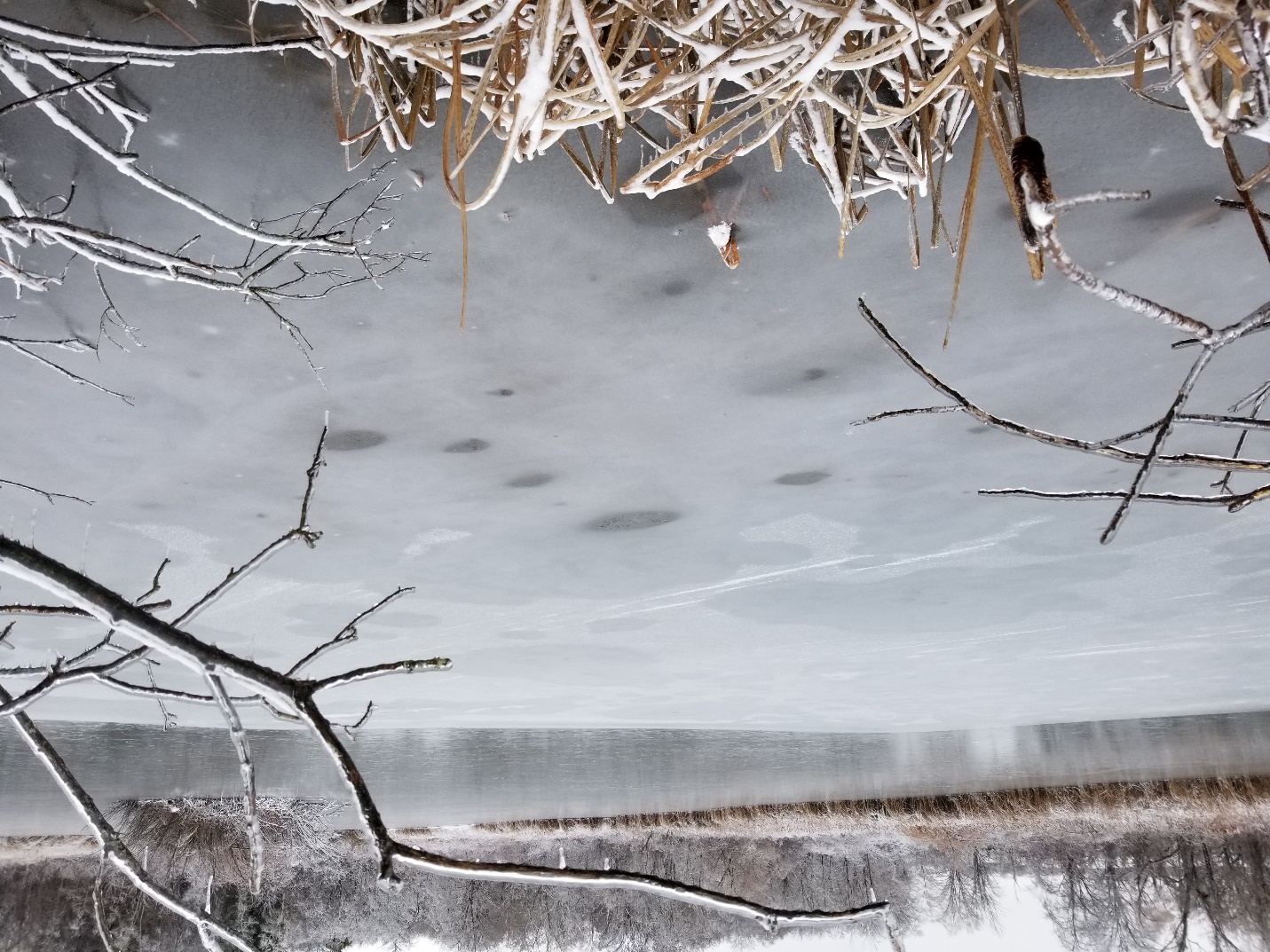


**Figure S15**. Picture showing ice holes during freeze over of the HB pond, signifying potential areas of preferential groundwater discharge.

Section B – Methodology details:

Groundwater sampling:

Sample handling:

Table S1. Details on water sample handling and storage. Full analyte lists in Table S2. Filtration was performed in the field with a 0.45-µm polyethersulfone membrane filter. Samples were kept in a cooler with ice packs in the field and during transport.

| **Analyte** | **Volume Collected / Bottle Type** | **Filtration** | **Preservation and Storage** |
| --- | --- | --- | --- |
| Dissolved metals + cations | 125ml polyethylene | Yes | pH < 2 with nitric acid 70% (M=15.8), stored in fridge 4℃ |
| Anions | 30ml polyethylene | Yes | Stored in fridge 4℃ |
| Ammonium | 30ml polyethylene | Yes | pH 5-6 with 10% hydrochloric acid; frozen until analyzed |
| Artificial Sweeteners | 30ml polyethylene | Yes | frozen until analyzed |
| Volatile Organic Compounds | 40ml glass with septa | No | pH <2 with NaHSO4, no headspace, stored in fridge 4℃ |
| Soluble Reactive Phosphorus | 40 ml glass | Yes | Stored in fridge 4℃ |
| Alkalinity | 125ml polyethylene | No | Stored in fridge 4℃ |

Table S2. List of compounds analyzed for each sampling suite.

| Artificial Sweeteners | Acesulfame, Saccharin, Cyclamate, Sucralose, Perchlorate, Glyphosate, 2,4-D, Fosamine, MCPA, Picloram, Sulfamic Acid |
| --- | --- |
| Anions | Fluoride, Chloride, Nitrite, Bromide, Sulfate, Nitrate, Phosphate |
| Cations | Calcium, Magnesium, Potassium, Silica, Sodium |
| Dissolved Metals | Antimony, Arsenic, Barium, Beryllium, Bismuth, Boron, Cadmium, Cerium, Cesium, Chromium, Cobalt, Copper, Gallium, Iron, Lanthanum, Lead, Lithium, Manganese, Molybdenum, Nickel, Niobium, Platinum, Rubidium, Selenium, Silver, Strontium, Thallium, Tin, Titanium, Tungsten, Uranium, Vanadium, Yttrium, Zinc |
| Volatile Organic Compounds | Chloromethane, vinyl chloride, bromomethane, chloroethane, diethyl ether, carbon disulfide, CFC-113, iodomethane, alyl chloride, methylene chloride, trans-1,2-dce, acetonitrile, chloropropene, 1,1-dichloroethane, acrylonitrile, cis-1,2-dce, dichloropropan, chloroform, carbon tetrachloride, 1,1,1-trichloroethane, THF, 1,1-dichloropropene, benzene, methylacrylonitrile, 1,2-dichloroethane, trichloroethene, dibromomethane, 1,2-dichlropropane, bromodichloromethane, methyl methacrylate, cis-1,3 dichloropropene, toluene, nitropropane, tetrachloroethene, trans-1,3-dichloropropene, 1,1,2--trichloroethane, ethyl methacrylate, dibromochloromethane, 1,3-dichlropropane, 1,2-dibromomethane, chlorobenzene, ethyl benzene, 1,1,1,2-tetrachloroethane, m+p-xylene, o-xylene, styrene, bromoform, isopropyl benzene, bromobenzene, polypropylbenzene, 1,1,2,2-tetrachloroethane, 2-chlorotoluene, 1,3,5-trimethylbenzene, 1,2,3-trichlropropane, trans-1,4-dichloro-2-butene, 4-chlorotoluene, tert-butylbenzene, pentachloroethane, 1,2,4-trimethylbenzene, sec-butylbenzene, p-cymene, 1,3-dichlorobenzene, 1,4-dichlorobenzene, n-butylbenzene, 1,2-dichlorobenzene, 1,2-dibromo-3-chloropropane, nitrobenzene, hexachlorobutadiene, 1,2,4-trichlorobenzene, naphthalene, 1,2,3-trichlorobenzene |

Chemical analyses:

All chemical analyses (full compound list in Table S2) were performed in Environment and Climate Change Canada laboratories at the Canada Centre for Inland Waters (Burlington, ON). Soluble reactive phosphorus (SRP) was measured with a Thermo Scientific Evolution 160 spectrophotometer using a mixed reagent of ammonium molybdenate and antimony potassium tartate (absorbance measured at 885nm). Ammonium was analyzed using a Beckman Coulter DU 720 general purpose spectrophotometer and a phenolhypochlorite reagent (absorbance measured at 640 nm). A set of 71 VOCs, largely chlorinated solvents and petroleum compounds, were analyzed with a Teledyne Tekmar Aquatek 70 autosampler, a Teledyne Tekmar 3100 sample concentrator purge and trap, an Agilent G1530A gas chromatograph, and a HP/Agilent 5973 mass selective detector. Anions, including chloride and nitrate, were analyzed with a Dionex 2500 ICS ion liquid chromatography system. Artificial sweeteners (saccharin and others) were analyzed The artificial sweeteners suite was determined with a Dionex 2500 ICS ion liquid chromatograph system combined with an Applied Biosystems AB Sciex QTrap 550 triple quad mass spectrometers (IC/MS/MS) with an electrospray ionization source in negative mode (IC/ESI/MS/MS). Direct sample injection of 100 μL was conducted onto a Dionex IONPAC® AS20 analytical column (2 × 250mm) with a KOH eluent gradient. Complete instrument details can be found in Van Stempvoort et al. (2020) with MRM details and compound specific parameters for saccharin reported in Van Stempvoort et al. (2019). Trace metals and cations were analyzed using Inductively Coupled Plasma-Sector Field Mass Spectrometry (ICP-MS, NLET method #2003) at the National Laboratory for Environmental Testing. Alkalinity was analyzed using HACH digital titration method 8203 with 1.6 N H_2_SO_4_.

Section C - Other data plots:


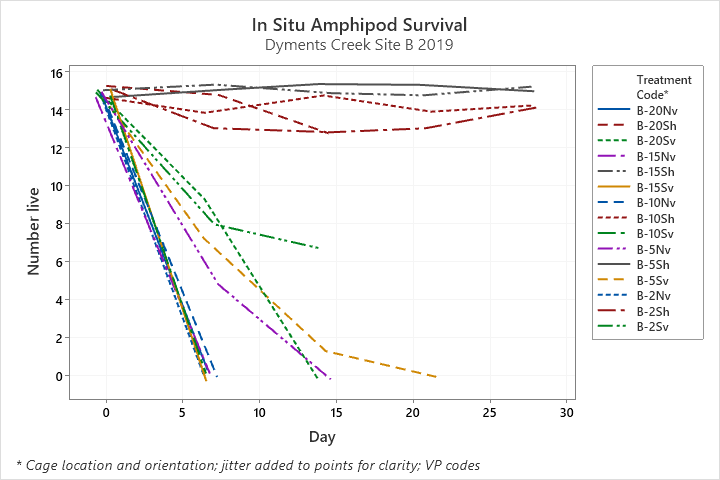


**Figure S16**. *Hyallela* survival for in situ cages placed at Stretch B of the DC site in 2019 (transects at 2, 5, 10, 15, 20 m (Fig. S7); v – vertical orientation; h – horizontal orientation).


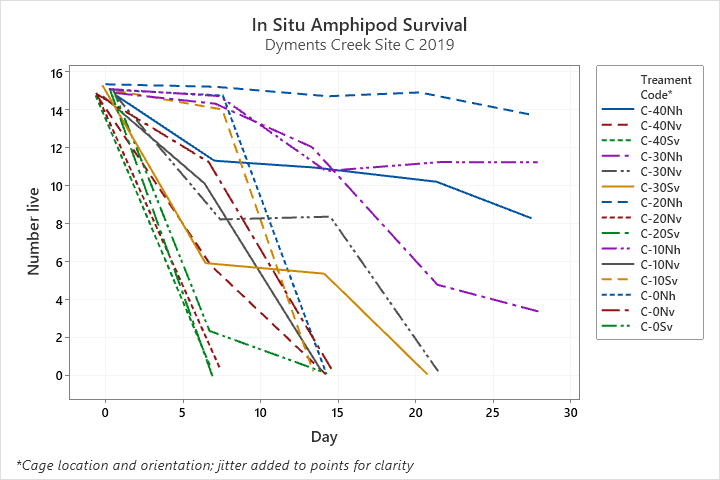


**Figure S17**. *Hyallela* survival for in situ cages placed at Stretch C of the DC site in 2019 (transects at 0, 10, 20, 30, 40 m (Fig. S7); v – vertical orientation; h – horizontal orientation).

**Figure S18**. Relationship between average mass of survivors for *Hyalella* cages of the 2022 Test 2 from the DC site.

**Figure S19**. Relationship between average mass of survivors for *Chironomus* cages of the 2022 Test 2 from the DC site.

Section D - Statistical data:

Highlights in blue indicate non-significance; highlights in yellow indicate significance (at 0.05 level)

1. **HRM Site:**

**Part 1 – Statistics associated with Fig. 2:**

**Median tests for differences between East and West, for specific orientation at each of the days. (HT – horizontal top; HB – horizontal half-buried; V – vertical)**

**Orientation HT, Day 2**

Median Test: Survival versus Location

Descriptive Statistics

| **Location** | **Median** | **N <= Overall Median** | **N > Overall Median** | **Q3 – Q1** | **95% Median CI** |
| --- | --- | --- | --- | --- | --- |
| East | 9.0 | 2 | 2 | 2.75 | (7, 10) |
| West | 8.5 | 2 | 2 | 3.25 | (6, 10) |
| Overall | 8.5 |  |  |  |  |

**Test**

| Null hypothesis | | | H₀: The population medians are all equal | |
| --- | --- | --- | --- | --- |
| Alternative hypothesis | | | H₁: The population medians are not all equal | |
| **DF** | **Chi-Square** | **P-Value** | |  |
| 1 | 0.00 | 1.000 | |  |

**Orientation HT, Day 4**

Median Test: Survival versus Location

Descriptive Statistics

| **Location** | **Median** | **N <= Overall Median** | **N > Overall Median** | **Q3 – Q1** | **95% Median CI** |
| --- | --- | --- | --- | --- | --- |
| East | 7 | 4 | 0 | 0.75 | (6, 7) |
| West | 8 | 2 | 2 | 3.50 | (6, 10) |
| Overall | 7 |  |  |  |  |

*Levels with < 6 observations have confidence < 95.0%
95.0% CI for median(East) - median(West): (-4,1)*

**Test**

| Null hypothesis | | | H₀: The population medians are all equal | |
| --- | --- | --- | --- | --- |
| Alternative hypothesis | | | H₁: The population medians are not all equal | |
| **DF** | **Chi-Square** | **P-Value** | |  |
| 1 | 2.67 | 0.102 | |  |

**Orientation HT, Day 7**

Median Test: Survival versus Location

Descriptive Statistics

| **Location** | **Median** | **N <= Overall Median** | **N > Overall Median** | **Q3 – Q1** | **95% Median CI** |
| --- | --- | --- | --- | --- | --- |
| East | 5.5 | 3 | 1 | 3.25 | (3, 7) |
| West | 7.5 | 1 | 3 | 3.25 | (5, 9) |
| Overall | 6.5 |  |  |  |  |

**Test**

| Null hypothesis | | | H₀: The population medians are all equal | |
| --- | --- | --- | --- | --- |
| Alternative hypothesis | | | H₁: The population medians are not all equal | |
| **DF** | **Chi-Square** | **P-Value** | |  |
| 1 | 2.00 | 0.157 | |  |

**Orientation HB, Day 3**

Median Test: Survival versus Location

Descriptive Statistics

| **Location** | **Median** | **N <= Overall Median** | **N > Overall Median** | **Q3 – Q1** | **95% Median CI** |
| --- | --- | --- | --- | --- | --- |
| East | 8.0 | 2 | 1 | 5 | (4, 9) |
| West | 9.0 | 1 | 2 | 2 | (7, 9) |
| Overall | 8.5 |  |  |  |  |

**Test**

| Null hypothesis | | | H₀: The population medians are all equal | |
| --- | --- | --- | --- | --- |
| Alternative hypothesis | | | H₁: The population medians are not all equal | |
| **DF** | **Chi-Square** | **P-Value** | |  |
| 1 | 0.67 | 0.414 | |  |

**Orientation HB, Day 6**

Median Test: Survival versus Location

Descriptive Statistics

| **Location** | **Median** | **N <= Overall Median** | **N > Overall Median** | **Q3 – Q1** | **95% Median CI** |
| --- | --- | --- | --- | --- | --- |
| East | 7 | 2 | 1 | 5 | (4, 9) |
| West | 9 | 1 | 2 | 2 | (7, 9) |
| Overall | 8 |  |  |  |  |

*Levels with < 6 observations have confidence < 95.0%
89.8% CI for median(East) - median(West): (-5,2)*

**Test**

| Null hypothesis | | | H₀: The population medians are all equal | |
| --- | --- | --- | --- | --- |
| Alternative hypothesis | | | H₁: The population medians are not all equal | |
| **DF** | **Chi-Square** | **P-Value** | |  |
| 1 | 0.67 | 0.414 | |  |

[Note: Chi-Square and P-value are same as in previous analysis]

**Orientation V, Day 2**

Median Test: Survival versus Location

Descriptive Statistics

| **Location** | **Median** | **N <= Overall Median** | **N > Overall Median** | **Q3 – Q1** | **95% Median CI** |
| --- | --- | --- | --- | --- | --- |
| East | 3.0 | 4 | 0 | 4.25 | (2, 7) |
| West | 9.5 | 0 | 4 | 1.75 | (8, 10) |
| Overall | 7.5 |  |  |  |  |

**Test**

| Null hypothesis | | | H₀: The population medians are all equal | |
| --- | --- | --- | --- | --- |
| Alternative hypothesis | | | H₁: The population medians are not all equal | |
| **DF** | **Chi-Square** | **P-Value** | |  |
| 1 | 8.00 | 0.005 | |  |

**Orientation V, Day 4**

Median Test: Survival versus Location

Descriptive Statistics

| **Location** | **Median** | **N <= Overall Median** | **N > Overall Median** | **Q3 – Q1** | **95% Median CI** |
| --- | --- | --- | --- | --- | --- |
| East | 1.5 | 3 | 1 | 3.25 | (1, 5) |
| West | 7.5 | 1 | 3 | 4.00 | (4, 9) |
| Overall | 4.5 |  |  |  |  |

**Test**

| Null hypothesis | | | H₀: The population medians are all equal | |
| --- | --- | --- | --- | --- |
| Alternative hypothesis | | | H₁: The population medians are not all equal | |
| **DF** | **Chi-Square** | **P-Value** | |  |
| 1 | 2.00 | 0.157 | |  |
|  |  |  | |  |

**Orientation V, Day 7**

Median Test: Survival versus Location

Descriptive Statistics

| **Location** | **Median** | **N <= Overall Median** | **N > Overall Median** | **Q3 – Q1** | **95% Median CI** |
| --- | --- | --- | --- | --- | --- |
| East | 1.5 | 4 | 0 | 1.00 | (1, 2) |
| West | 5.0 | 0 | 4 | 2.75 | (4, 7) |
| Overall | 3.0 |  |  |  |  |

**Test**

| Null hypothesis | | | H₀: The population medians are all equal | |
| --- | --- | --- | --- | --- |
| Alternative hypothesis | | | H₁: The population medians are not all equal | |
| **DF** | **Chi-Square** | **P-Value** | |  |
| 1 | 8.00 | 0.005 | |  |

**Part 2 – Statistics associated with Fig. 3 – survivor mass data**

**Comparison of median Dry Weight of surviving Hyalella in 6 cage treatment groups**

1. Kruskal-Wallis Test:

Descriptive Statistics

| **Cage Trt** | **N** | **Median** | **Mean Rank** | **Z-Value** |
| --- | --- | --- | --- | --- |
| E-HB | 3 | 0.174286 | 6.7 | -1.39 |
| E-HT | 4 | 0.224333 | 15.3 | 1.28 |
| E-V | 4 | 0.245000 | 15.0 | 1.19 |
| W-HB | 3 | 0.177500 | 9.7 | -0.53 |
| W-HT | 4 | 0.175357 | 7.0 | -1.53 |
| W-V | 4 | 0.220893 | 13.8 | 0.77 |
| Overall | 22 |  | 11.5 |  |

Test

| Null hypothesis | | | H₀: All medians are equal | |
| --- | --- | --- | --- | --- |
| Alternative hypothesis | | | H₁: At least one median is different | |
| **DF** | **H-Value** | **P-Value** | |  |
| 5 | 6.80 | 0.236 | |  |

*The chi-square approximation may not be accurate when some sample sizes are less than 5.*

1. Median test: **Hyallela**

Descriptive Statistics

| **Cage Trt** | **Median** | **N <= Overall Median** | **N > Overall Median** | **Q3 – Q1** | **95% Median CI** |
| --- | --- | --- | --- | --- | --- |
| E-HT | 0.224333 | 1 | 3 | 0.0338452 | (0.211429, 0.248333) |
| W-HT | 0.175357 | 3 | 1 | 0.0666548 | (0.132, 0.214444) |
| Overall | 0.211714 |  |  |  |  |

*Levels with < 6 observations have confidence < 95.0%
95.0% CI for median(E-HT) - median(W-HT): (-0.00301587,0.116333)*

Test

| Null hypothesis | | | H₀: The population medians are all equal | |
| --- | --- | --- | --- | --- |
| Alternative hypothesis | | | H₁: The population medians are not all equal | |
| **DF** | **Chi-Square** | **P-Value** | |  |
| 1 | 2.00 | 0.157 | |  |

Descriptive Statistics

| **Cage Trt** | **Median** | **N <= Overall Median** | **N > Overall Median** | **Q3 – Q1** | **95% Median CI** |
| --- | --- | --- | --- | --- | --- |
| E-HB | 0.174286 | 2 | 1 | 0.0441667 | (0.163333, 0.2075) |
| W-HB | 0.177500 | 1 | 2 | 0.0562500 | (0.17375, 0.23) |
| Overall | 0.175893 |  |  |  |  |

*Levels with < 6 observations have confidence < 95.0%
89.8% CI for median(E-HB) - median(W-HB): (-0.0666667,0.03375)*

Test

| Null hypothesis | | | H₀: The population medians are all equal | |
| --- | --- | --- | --- | --- |
| Alternative hypothesis | | | H₁: The population medians are not all equal | |
| **DF** | **Chi-Square** | **P-Value** | |  |
| 1 | 0.67 | 0.414 | |  |

Descriptive Statistics

| **Cage Trt** | **Median** | **N <= Overall Median** | **N > Overall Median** | **Q3 – Q1** | **95% Median CI** |
| --- | --- | --- | --- | --- | --- |
| E-V | 0.245000 | 2 | 2 | 0.210000 | (0.16, 0.42) |
| W-V | 0.220893 | 2 | 2 | 0.074554 | (0.175, 0.256667) |
| Overall | 0.231250 |  |  |  |  |

*Levels with < 6 observations have confidence < 95.0%
95.0% CI for median(E-V) - median(W-V): (-0.0966667,0.245)*

Test

| Null hypothesis | | | H₀: The population medians are all equal | |
| --- | --- | --- | --- | --- |
| Alternative hypothesis | | | H₁: The population medians are not all equal | |
| **DF** | **Chi-Square** | **P-Value** | |  |
| 1 | 0.00 | 1.000 | |  |

1. **HB Site:**

**Statistics associated with Fig. 4:**

Within plume samples (1) – locations 1-9

Outside of plume samples (0) – locations 10-15


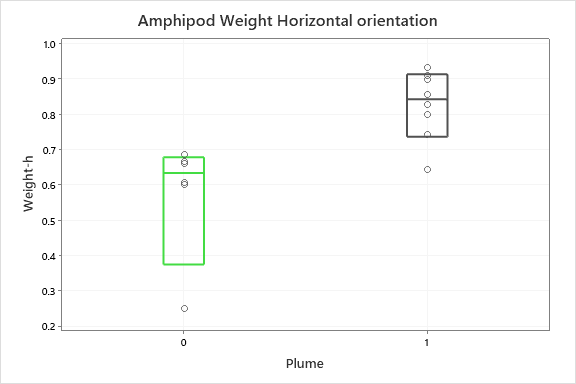


**Figure S20**. Weights from most plume stations with cages in horizontal position were higher than cages not in the plume.


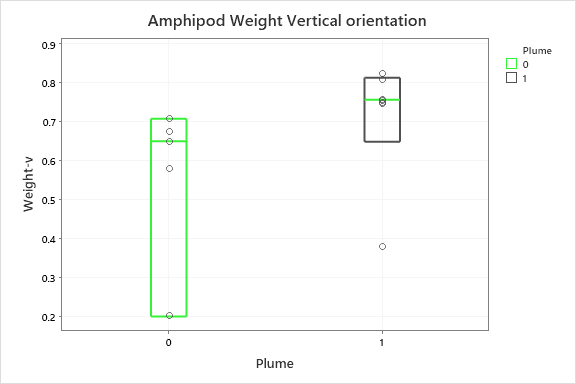


**Figure S21**. Weights from most plume stations with cages in vertical position were higher than cages not in the plume.

**Median tests showing** **Survivor-horizontal and** **Survivor- vertical v. plume.**

**Descriptive Statistics for Survivor-horizontal**

| **Plume** | **Median** | **N <= Overall Median** | **N > Overall Median** | **Q3 – Q1** | **95% Median CI** |
| --- | --- | --- | --- | --- | --- |
| 0 | 12.5 | 4 | 2 | 5.25 | (9.35714, 15) |
| 1 | 13.5 | 4 | 4 | 3.75 | (10.8069, 15) |
| Overall | 13.0 |  |  |  |  |

*95.0% CI for median(0) - median(1): (-5.1303,3.18783)*

**Test for Survivor-horizontal**

| Null hypothesis | | | H₀: The population medians are all equal | |
| --- | --- | --- | --- | --- |
| Alternative hypothesis | | | H₁: The population medians are not all equal | |
| **DF** | **Chi-Square** | **P-Value** | |  |
| 1 | 0.39 | 0.533 | |  |

**Descriptive Statistics for Survivor- vertical**

| **Plume** | **Median** | **N <= Overall Median** | **N > Overall Median** | **Q3 – Q1** | **95% Median CI** |
| --- | --- | --- | --- | --- | --- |
| 0 | 13.0 | 3 | 2 | 8.00 | (1, 15) |
| 1 | 13.5 | 4 | 4 | 1.75 | (11.2277, 14.1287) |
| Overall | 13.0 |  |  |  |  |

*Levels with < 6 observations have confidence < 95.0%
95.0% CI for median(0) - median(1): (-13,2.12326)*

**Test for Survivor- vertical**

| Null hypothesis | | | H₀: The population medians are all equal | |
| --- | --- | --- | --- | --- |
| Alternative hypothesis | | | H₁: The population medians are not all equal | |
| **DF** | **Chi-Square** | **P-Value** | |  |
| 1 | 0.12 | 0.725 | |  |

No difference in Survivor medians in plume v. out of plume.

**Median tests showing Growth-horizontal and Growth- vertical v. plume.**

**Descriptive Statistics for Weight-horizontal**

| **Plume** | **Median** | **N <= Overall Median** | **N > Overall Median** | **Q3 – Q1** | **95% Median CI** |
| --- | --- | --- | --- | --- | --- |
| 0 | 0.634167 | 6 | 0 | 0.158929 | (0.375, 0.678912) |
| 1 | 0.843277 | 1 | 7 | 0.152232 | (0.736479, 0.913841) |
| Overall | 0.714286 |  |  |  |  |

*95.0% CI for median(0) - median(1): (-0.347952,-0.120118)*

**Test for Weight-horizontal**

| Null hypothesis | | | H₀: The population medians are all equal | |
| --- | --- | --- | --- | --- |
| Alternative hypothesis | | | H₁: The population medians are not all equal | |
| **DF** | **Chi-Square** | **P-Value** | |  |
| 1 | 10.50 | 0.001 | |  |

**Descriptive Statistics** **for Weight-vertical**

| **Plume** | **Median** | **N <= Overall Median** | **N > Overall Median** | **Q3 – Q1** | **95% Median CI** |
| --- | --- | --- | --- | --- | --- |
| 0 | 0.650000 | 5 | 0 | 0.301346 | (0.2, 0.707692) |
| 1 | 0.756250 | 1 | 6 | 0.061667 | (0.648889, 0.812778) |
| Overall | 0.727179 |  |  |  |  |

*Levels with < 6 observations have confidence < 95.0%
95.0% CI for median(0) - median(1): (-0.608333,-0.0389744)*

**Test for Weight-vertical**

| Null hypothesis | | | H₀: The population medians are all equal | |
| --- | --- | --- | --- | --- |
| Alternative hypothesis | | | H₁: The population medians are not all equal | |
| **DF** | **Chi-Square** | **P-Value** | |  |
| 1 | 8.57 | 0.003 | |  |

Average mass is higher in the plume than out of the plume.

**Correlation plots:**


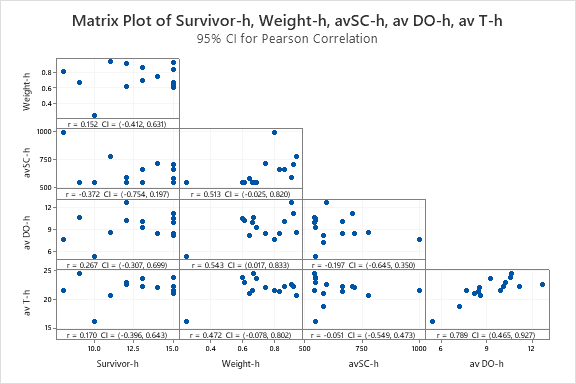


**Figure S22**. Pearson Correlations for horizontal cages (*Hyallela*): survivors, average survivor mass (weight), average in-cage specific conductance (SC), dissolved oxygen (DO) and temperature (T).


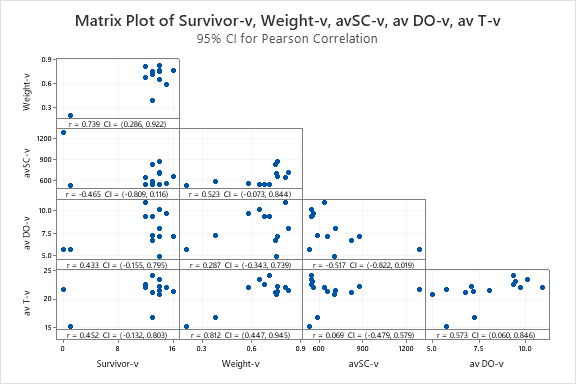


**Figure S23**. Pearson Correlations for vertical cages (*Hyallela*): survivors, average survivor mass (weight), average in-cage specific conductance (SC), dissolved oxygen (DO) and temperature (T).

1. **DC Site in 2019**

**Table S3**. Pearson and Spearman correlations results between 7-day survival for *Hyallela* at the DC site in 2019 (Study 3) and groundwater contaminants / properties. (negative values indicate potential toxic effect)

| Contaminant / Property | Pearson r | Spearman r_s_ |
| --- | --- | --- |
| Specific Conductivity | -0.003 | -0.170 |
| Dissolved Oxygen | -0.314 | -0.299 |
| Saccharin | -0.262 | 0.015 |
| Cyclamate | -0.156 | -0.104 |
| Acesulfame | 0.623 | 0.582 |
| Sucralose | 0.597 | 0.580 |
| NH_4_^+^ | -0.167 | 0.174 |
| Cl | -0.514 | -0.589 |
| chloromethane | 0.452 | 0.340 |
| vinyl chloride | 0.063 | -0.005 |
| bromomethane | 0.230 | 0.176 |
| chloroethane | 0.292 | 0.256 |
| diethylether | -0.090 | 0.128 |
| carbon disulfide | -0.410 | -0.476 |
| iodomethane | 0.103 | 0.080 |
| methylene chloride | 0.266 | 0.203 |
| trans-1,2-dce | -0.329 | -0.382 |
| acetonitrile | -0.146 | -0.198 |
| 1,1-dichloroethane | -0.087 | -0.091 |
| cis-1,2-dce | -0.221 | -0.222 |
| THF | -0.242 | -0.247 |
| benzene | 0.125 | 0.290 |
| 1,2-dichloroethane | -0.102 | 0.017 |
| trichloroethene | -0.183 | 0.044 |
| toluene | 0.342 | 0.430 |
| tetrachloroethene | -0.219 | -0.230 |
| chlorobenzene | -0.075 | 0.169 |
| ethyl benzene | 0.072 | 0.236 |
| m+p-xylene | -0.065 | 0.335 |
| o-xylene | 0.123 | 0.361 |
| BTEX | 0.077 | 0.367 |
| isopropyl benzene | 0.154 | 0.150 |
| polypropylbenzene | 0.108 | 0.346 |
| 1,3,5-trimethylbenzene | 0.244 | 0.382 |
| 1,2,4-trimethylbenzene | 0.072 | 0.153 |
| sec-butylbenzene | -0.117 | -0.021 |
| p-cymene | 0.052 | 0.148 |
| 1,3-dichlorobenzene | 0.029 | -0.153 |
| 1,4-dichlorobenzene | 0.013 | 0.115 |
| n-butylbenzene | -0.045 | -0.080 |
| 1,2-dichlorobenzene | 0.017 | 0.017 |
| Sum Sub Benz | 0.058 | 0.212 |
| naphthalene | 0.122 | 0.170 |
| Al | 0.399 | 0.398 |
| B | 0.011 | -0.150 |
| Fe | 0.126 | 0.189 |
| As | 0.192 | 0.191 |
| Ba | 0.095 | 0.319 |
| Cr | 0.255 | 0.105 |
| Cu | -0.129 | 0.448 |
| Li | -0.095 | 0.284 |
| Mn | 0.101 | -0.122 |
| Zn | -0.054 | 0.251 |
| Nickel | 0.042 | 0.201 |
| Selenium | 0.081 | 0.256 |
| Strontium | 0.098 | 0.157 |
| Titanium | 0.214 | 0.318 |
| Vanadium | -0.084 | 0.036 |
| Antimony | 0.199 | 0.272 |
| Beryllium | 0.105 | 0.422 |
| Bismuth | 0.188 | 0.363 |
| Cadmium | -0.183 | 0.096 |
| Cerium | -0.026 | 0.073 |
| Cesium |  | 0.319 |
| Cobalt |  | 0.315 |
| Gallium |  | 0.103 |
| Lanthanum |  | 0.128 |
| Lead |  | 0.369 |
| Molybdenum |  | 0.341 |
| Niobium |  | 0.330 |
| Rubidium |  | 0.245 |
| Silver |  | 0.098 |
| Tin |  | 0.094 |
| Tungsten |  | 0.365 |
| Yttrium |  | 0.108 |
| Uranium |  | 0.084 |
|  |  |  |

1. **DC Site in 2022 – Test 1**

Median tests - North side of stream is impacted by landfill leachate. South side is comparatively not impacted.

**Descriptive Statistics: Hyalella**

| **Stream Side_Amphipod** | **Median** | **N <= Overall Median** | **N > Overall Median** | **Q3 – Q1** | **95% Median CI** |
| --- | --- | --- | --- | --- | --- |
| North | 0.0 | 4 | 0 | 0.0 | (0, 0) |
| South | 9.5 | 0 | 4 | 2.5 | (7, 10) |
| Overall | 3.5 |  |  |  |  |

**Test**

| Null hypothesis | | | H₀: The population medians are all equal | |
| --- | --- | --- | --- | --- |
| Alternative hypothesis | | | H₁: The population medians are not all equal | |
| **DF** | **Chi-Square** | **P-Value** | |  |
| 1 | 8.00 | 0.005 | |  |

**Descriptive Statistics: Chironomus**

| **Stream Side_Midge** | **Median** | **N <= Overall Median** | **N > Overall Median** | **Q3 – Q1** | **95% Median CI** |
| --- | --- | --- | --- | --- | --- |
| North | 0 | 4 | 0 | 0.0 | (0, 0) |
| South | 4 | 2 | 2 | 9.5 | (0, 10) |
| Overall | 0 |  |  |  |  |

**Test**

| Null hypothesis | | | H₀: The population medians are all equal | |
| --- | --- | --- | --- | --- |
| Alternative hypothesis | | | H₁: The population medians are not all equal | |
| **DF** | **Chi-Square** | **P-Value** | |  |
| 1 | 2.67 | 0.102 | |  |

1. **DC Site in 2022 – Test 2**

**Statistics associated with Fig. 6:**

Median Tests - North side of stream is impacted by landfill leachate. South side is comparatively not impacted.

**Descriptive Statistics: Hyalella**

| **Stream Side_Amphipod** | **Median** | **N <= Overall Median** | **N > Overall Median** | **Q3 – Q1** | **95% Median CI** |
| --- | --- | --- | --- | --- | --- |
| North | 0.0 | 7 | 1 | 1.00 | (0, 1.45050) |
| South | 8.0 | 1 | 7 | 5.75 | (1.87129, 9.06436) |
| Overall | 1.5 |  |  |  |  |

*95.0% CI for median(North) - median(South): (-9,-4.49533)*

**Test: Hyalella**

| Null hypothesis | | | H₀: The population medians are all equal | |
| --- | --- | --- | --- | --- |
| Alternative hypothesis | | | H₁: The population medians are not all equal | |
| **DF** | **Chi-Square** | **P-Value** | |  |
| 1 | 9.00 | 0.003 | |  |

**Descriptive Statistics: Chironomus**

| **Stream Side_Midge** | **Median** | **N <= Overall Median** | **N > Overall Median** | **Q3 – Q1** | **95% Median CI** |
| --- | --- | --- | --- | --- | --- |
| North | 4.0 | 7 | 1 | 5.50 | (0.935644, 7.12871) |
| South | 9.5 | 2 | 6 | 2.75 | (6.87129, 10) |
| Overall | 7.0 |  |  |  |  |

*95.0% CI for median(North) - median(South): (-7.60187,-0.699065)*

**Test: Chironomus**

| Null hypothesis | | | H₀: The population medians are all equal | |
| --- | --- | --- | --- | --- |
| Alternative hypothesis | | | H₁: The population medians are not all equal | |
| **DF** | **Chi-Square** | **P-Value** | |  |
| 1 | 6.35 | 0.012 | |  |

**Regression assessment for survival data from DC Site – 2022 for Hyallela and Chironomus against landfill indicators saccharin and ammonium-N**

The regression equation is
Hyalella S = 6.925 - 0.001847 Saccharin

**Model Summary**

| **S** | **R-sq** | **R-sq(adj)** |
| --- | --- | --- |
| 2.93363 | 54.99% | 47.49% |

**Analysis of Variance**

| **Source** | **DF** | **SS** | **MS** | **F** | **P** |
| --- | --- | --- | --- | --- | --- |
| Regression | 1 | 63.082 | 63.0817 | 7.33 | 0.035 |
| Error | 6 | 51.637 | 8.6062 |  |  |
| Total | 7 | 114.719 |  |  |  |


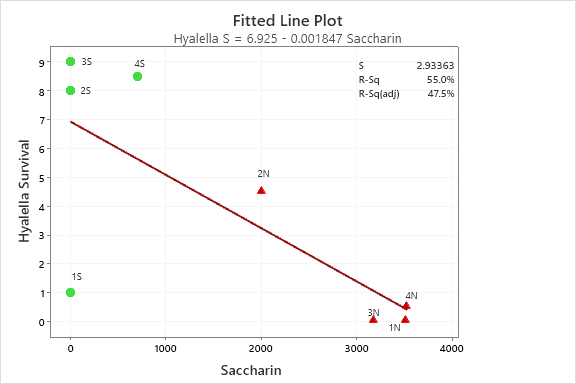


**Figure S24**. Regression analysis of relationship between saccharin concentration and *Hyallela* survival.

The regression equation is
Hyalella S = 6.774 - 0.04782 NH4[N] (mg/L)

**Model Summary**

| **S** | **R-sq** | **R-sq(adj)** |
| --- | --- | --- |
| 2.76064 | 60.14% | 53.50% |

**Analysis of Variance**

| **Source** | **DF** | **SS** | **MS** | **F** | **P** |
| --- | --- | --- | --- | --- | --- |
| Regression | 1 | 68.992 | 68.9918 | 9.05 | 0.024 |
| Error | 6 | 45.727 | 7.6212 |  |  |
| Total | 7 | 114.719 |  |  |  |


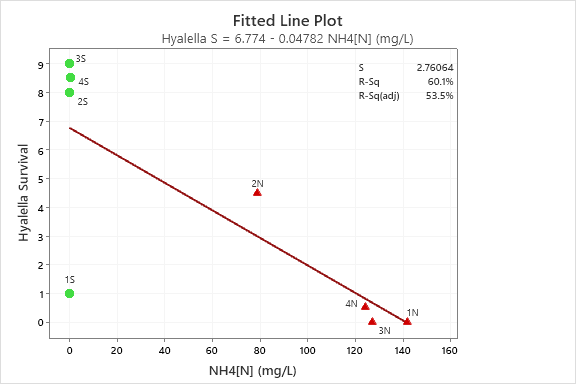


**Figure S25**. Regression analysis of relationship between ammonium-N concentration and *Hyallela* survival.

The regression equation is
Chironomus S = 9.177 - 0.001655 Saccharin

**Model Summary**

| **S** | **R-sq** | **R-sq(adj)** |
| --- | --- | --- |
| 1.74984 | 73.37% | 68.94% |

**Analysis of Variance**

| **Source** | **DF** | **SS** | **MS** | **F** | **P** |
| --- | --- | --- | --- | --- | --- |
| Regression | 1 | 50.6284 | 50.6284 | 16.53 | 0.007 |
| Error | 6 | 18.3716 | 3.0619 |  |  |
| Total | 7 | 69.0000 |  |  |  |


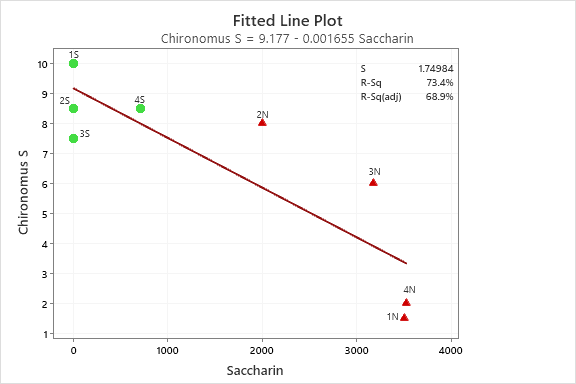


**Figure S26**. Regression analysis of relationship between saccharin concentration and *Chironomus* survival.

The regression equation is
Chironomus S = 8.890 - 0.04029 NH4[N] (mg/L)

**Model Summary**

| **S** | **R-sq** | **R-sq(adj)** |
| --- | --- | --- |
| 1.82660 | 70.99% | 66.15% |

**Analysis of Variance**

| **Source** | **DF** | **SS** | **MS** | **F** | **P** |
| --- | --- | --- | --- | --- | --- |
| Regression | 1 | 48.9812 | 48.9812 | 14.68 | 0.009 |
| Error | 6 | 20.0188 | 3.3365 |  |  |
| Total | 7 | 69.0000 |  |  |  |


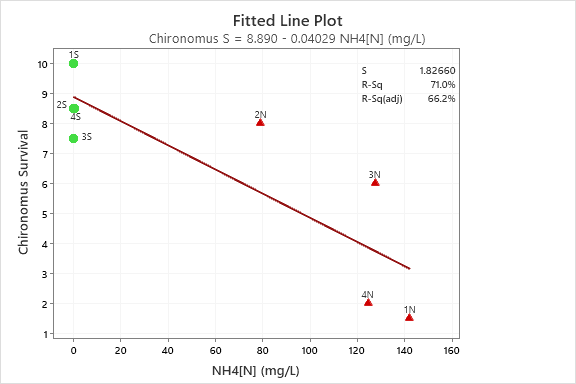


**Figure S27**. Regression analysis of relationship between ammonium-N concentration and *Chironomus* survival.

**PCA of DC Site 2022 Test 2**


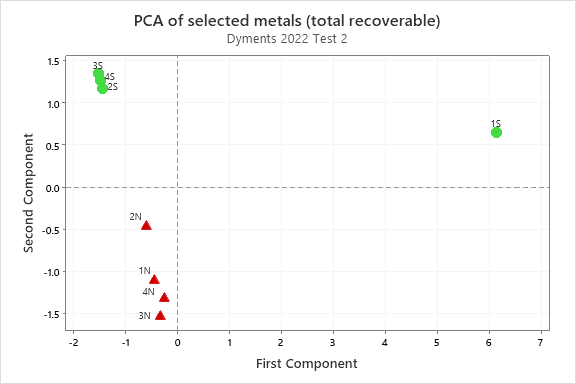


**Figure S28**. Principal Components Analysis (PCA) of toxicologically important metal concentrations for discharging groundwater samples from the 2022 DC Site study (4 from N side (landfill affected), red solid triangles; 4 from S side (not affected), green solid circles).


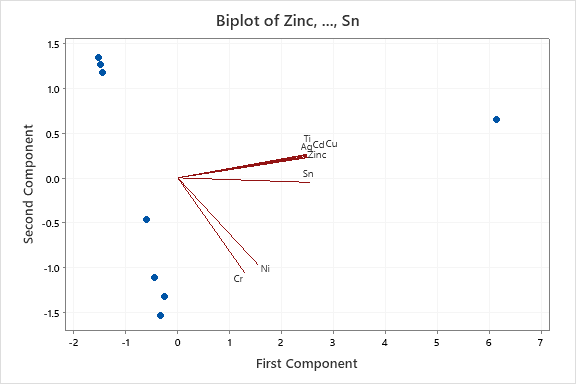


**Figure S29**. PCA biplot of first two components for metal concentrations (shown as vectors) for discharging groundwater samples (see Fig. S28) from the 2022 DC Site study. Percent variance explained is 80.5% and 19.2% for components 1 and 2, respectively. Sample scores are shown unlabeled.

References:

Hua T, Propp VR, Power C, Brown, SJ, Collins, P, Smith, JE and Roy, JW, (2023) Multi‐zone aquatic ecological exposures to landfill contaminants from a groundwater plume discharging to a pond. Environ Toxicol Chem*.* https://doi.org/10.1002/etc.5650

Van Stempvoort DR, MacKay RD, Brown SJ, Collins P (2020) Environmental fluxes of perchlorate in rural catchments, Ontario, Canada. Sci Total Environ 720. DOI: 10.1016/j.scitotenv.2020.137426

Van Stempvoort, DR, Spoelstra J, Brown SJ, Post R, Smyth SA (2019) Sulfamate in environmental waters. Sci Total Environ 695. DOI: 10.1016/j.scitotenv.2019.133734
